# Comprehensive assessment of candidate genes associated with fattening performance in Holstein–Friesian bulls

**DOI:** 10.5194/aab-62-9-2019

**Published:** 2019-01-21

**Authors:** Sena Ardicli, Hale Samli, Buse Vatansever, Bahadir Soyudal, Deniz Dincel, Faruk Balci

**Affiliations:** 1 Laboratory of Genetics, Department of Genetics, Faculty of Veterinary Medicine, Uludag University, 16059 Nilufer, Bursa, Turkey; 2 Department of Biology, Institute of Science, Uludag University, 16059 Nilufer, Bursa, Turkey

## Abstract

The objective of this study was to determine the association of single nucleotide polymorphisms (SNPs) in selected candidate genes with fattening performance traits in a commercial cattle herd. Fifteen SNPs in 12 candidate genes (*LEP*, *FABP4*, *DGAT1*, *TG*, *IGF1*, *IGF1R*, *MYF5*, *LGB*, *CAPN1*, *CAST*, *GHR*, and *OLR1*) were evaluated in 296 purebred Holstein–Friesian bulls using PCR-RFLP (polymerase chain reaction – restriction fragment length polymorphism). Associations between each segregating SNP and genetic merit for fattening performance were quantified using linear mixed models. Traits included in the study were fattening period, final weight, dry matter intake, feed conversion rate, and average daily weight gain. Apart from the general determination of the above-mentioned traits, each trait was evaluated based on the fattening periods between five selected target body weights (W1 = 100 kg, W2 = 200 kg, W3 = 300 kg, W4 = 400 kg, W5 = 450 kg). All markers with the exception of *CAPN1* 530, *IGF1R*, *TG*, and *DGAT1* were associated with at least one of the traits. Furthermore, novel associations were observed for *LEP* × *GHR*, *IGF1* × *LEP*, *FABP4* 3691 × *FABP4* 2834, and *FAP4* 3533 × *LEP* interactions. The results of this study confirm some previously reported associations. Moreover, novel associations have been identified, which may be incorporated into breeding programs to improve fattening performance.

## 1 Introduction

Several methods of evaluating fattening performance have been presented over the past few decades. In cattle fattening farms, techno-economic performance optimization is imperative for reaching an adequate profitability, whether in calf-to-finish or fattening farms. Prior knowledge regarding the commercial targets allows for the determination of objectives such as slaughter weight, weight gain, feed consumption, and meat quality. Improved breeding methods as well as the application of biotechnology have advanced the efficiency of cattle production (Pfuhl et al., 2007). Achieving satisfactory fattening performance and profitability are affected by breed of the animals, season, initial weight, concentrate level, sex, penned cattle population, and housing type; and in addition to this, they are closely associated with optimal slaughter ages and final weights, which vary widely among cattle breeds (Koknaroglu et al., 2005; Alberti et al., 2008). Apart from these environmental factors, the genotypic structure of the animals is another decisive constituent of an efficient fattening performance evaluation in cattle production, which necessitates a long generation interval.

Recently, many pieces of evidence have been presented that show that fattening performance and carcass traits are rather influenced by a number of candidate genes in various cattle breeds (Oprzadek and Flisikowski, 2003; Maj et al., 2004; Curi et al., 2005a). The bovine leptin gene (*LEP*; GenBank accession number: AF536174.1), which is located on chromosome 4, regulates food intake and whole-body energy metabolism (Nkrumah et al., 2005; Banos et al., 2008). In addition, this gene is also involved in the regulation of body weight and growth traits (Lusk, 2007; Kulig and Kmiec, 2009), dry matter intake (Banos et al., 2008), and therefore it may be considered as one of the most effective biological markers reflecting body fatness (Oprzadek and Flisikowski, 2003; Shin and Chung, 2007a; Corva et al., 2009). Similar to the *LEP*, the bovine fatty acid binding protein 4 (*FABP4*) gene plays an important role in lipid metabolism associated with changes in lipid hydrolysis and intracellular fatty acid (Casas et al., 2003). *FABP4* is a functional and positional candidate gene for fat synthesis in cattle (Shin and Chung, 2007a; Fortes et al., 2009). Bovine chromosome 14 (BTA14), where *FABP4* is located, is widely known to harbor quantitative trait loci (QTL) associated with fat-related traits such as milk fat percentage (Grisart et al., 2002), back fat thickness (Moore et al., 2003), and marbling (Ardicli et al., 2017b). The other important markers that have been mapped to BTA14 are diacylglycerol-O-acyltransferase 1 (*DGAT1*) and thyroglobulin (*TG*). *DGAT1* (GenBank accession number: AY065621) has been shown to be commonly associated with milk components and intramuscular fat content (Grisart et al., 2002; Hradecka et al., 2008; Curi et al., 2011). *TG* (GenBank accession number: X05380) is a glycoprotein precursor and the molecular regulator for the thyroid hormones. This gene has been demonstrated to be associated with lipid metabolism and meat production traits in various cattle breeds (Barendse et al., 2004; Burrell et al., 2004; Shin and Chung, 2007b).

Bovine chromosome 5 (BTA5) harbors QTLs that influence milk production (Kalm et al., 1998), reproduction (Kirkpatrick et al., 2000), and growth and carcass traits (Stone et al., 1999; Casas et al., 2000; Li et al., 2004). In this genomic region, the location of some of the QTLs approaches the position of the insulin-like growth factor (*IGF1*) and myogenic factor 5 (*MYF5*) genes that play fundamental roles in regulation of growth and development (Machado et al., 2003; Kisacova et al., 2009). *IGF1* (GenBank accession number: AF210383) has been shown to be a strong candidate gene for growth rate and meat production traits (Machado et al., 2003; Li et al., 2004; Curi et al., 2005a; Siadkowska et al., 2006) owing to its key role in regulation of cell proliferation (Siadkowska et al., 2006). In addition to *IGF1*, its associated receptor *IGF1R *(GenBank accession number: U33122), plays an important role in the regulation of the metabolism and physiology of mammalian growth (Curi et al., 2005a). *MYF5* (GenBank Accession number: M95684), which is a member of the muscle regulatory factors (*MRF*) gene family, was determined as a muscle-specific factor and its expression was associated with myoblast lineage. Polymorphisms in this gene have been reported to be effective on growth traits in cattle (Chung and Kim, 2005; Zhang et al., 2007). The bovine oxidized low-density lipoprotein receptor 1 (*OLR1*) gene was mapped in the interval of 106 to 108 cM (centimorgan) on BTA 5 (Khatib et al., 2006). Several studies with dairy cattle had shown significant effects of the *OLR1* marker on milk production traits (Khatib et al., 2006; Komisarek and Dorynek, 2009). On the other hand, the in vivo physiological role of *OLR1* in metabolism was reported by Murase et al. (2000) and Vinsky et al. (2013).

The beta-lactoglobulin (*LGB*) gene, situated on bovine chromosome 11, encodes the main protein of whey (Tambasco et al., 2003). *LGB* (GenBank accession number: X14710) locus plays a key role in the evaluation of the milk production potential, and in addition this gene can also be associated with other loci that have a direct influence on growth (Curi et al., 2005b).

The activity of the calpain–calpastatin proteolytic system is closely related to meat quality through postmortem tenderization and is characterized by three components including μ-calpain, m-calpain, and calpastatin, which is a specific endogenous protease inhibitor of calpains (Curi et al., 2009). In addition to this, μ-calpain and calpastatin are encoded by the μ-calpain (*CAPN1*; GenBank accession number: AF252504) and calpastatin (*CAST*; GenBank accession number: AF117813) genes, located on bovine chromosomes 29 and 7, respectively (Corva et al., 2007; Curi et al., 2009).

Growth hormone receptor gene (*GHR*), mapped to bovine chromosome 20, encodes growth hormone (GH), also known as somatotrophin, which stimulates important physiological processes involving growth and metabolism in cattle (Di Stasio et al., 2005; Sherman et al., 2008).

Over the last few decades, beef production in Turkey has become one of the most important activities in terms of generating wealth and employment for the country. In 2017, a total of 1 126 403 t of red meat was produced from 10 811 442 animals in Turkey. This production originated from 3 602 115 cattle, 5 134 338 sheep, 2 068 866 goats, and 6123 water buffaloes. The most significant part of this production derived from beef with a total of 987 482 t (93.16 % of total; Turkish Statistical Institute, 2018). However, the current production needs to be more efficient to supply internal demands. Increased demand and insufficient production have resulted in high meat prices. Thus, Turkish ministries have allowed for the importation of living animals and carcasses to regulate supply and demand but the importation has not been effective in lowering price increases (Ustuner et al., 2017). In Turkey, there is a strong need for novel and analytical breeding strategies, including methods for genome-assisted prediction of quantitative traits. These strategies may help with maintaining or increasing production and help Turkey become a self-sufficient country with respect to beef production. In many countries, meat production originates from two sources: production from herds that are exclusively based on specific beef breeds and production from a combination of dairy or meat products. Cattle farms in Turkey generally produce dairy and dual-purpose breeds, and the number of specific beef breeds is rather limited. In this respect, the most common cattle raised in Turkey (43.84 %) is the Holstein breed, with 6 989 126 purebreds and crosses. Considering 15 943 586 total cattle count, the Holstein breed has a significant impact not only on the dairy cattle but also on Turkish beef production (Turkish Statistical Institute, 2018). Therefore, evaluating the potential of Holstein meat should be considered to increase beef production in the country.

Holstein cattle, which exhibit excellent dairy-type characteristics, carry a potential for covering the shortage in meat production by means of genetic variability for beef traits and therefore the dual capacity of the Holstein breed is used in several countries for improvement of beef production (Calo et al., 1973). However, there is limited information about the associations between the genotypic structure and growth of fattening performance traits in Holsteins. Therefore, the main purpose of this paper is to obtain a detailed perspective on the effects of 15 polymorphisms at the selected candidate genes *LEP*, *FABP4*, *DGAT1*, *TG*, *IGF1*, *IGF1R*, *MYF5*, *LGB*, *CAPN1*, *CAST*, *GHR*, and *OLR1* on fattening performance in Holstein bulls. In addition, the combined effects of these markers were evaluated with respect to genotypic interactions.

## 2 Materials and methods

### 2.1 Animals, management, and determination of fattening performance

The animals used in this study were recorded for the Pedigree Project of the Turkish Ministry of Food, Agriculture and Livestock, and Cattle Breeders Association. Ethical approval for this study was granted by the Uludag University local Research Ethics Committee (approval number: 2017-05/06). A total of 296 Holstein–Friesian bulls that were randomly selected from a commercial herd (with a herd size of 10 000 cattle) and raised on the same farm located in the south Marmara region of Turkey (40${}^{\circ }$18${}^{\prime }$06.0${}^{\prime \prime }$ N, 27${}^{\circ }$56${}^{\prime }$28.5${}^{\prime \prime }$ E) were used in the present study. Maximum and minimum ambient air temperatures (${}^{\circ }$C) recorded in the sheds during the period of the study were 16.83±2.20 and 7.43±1.10${}^{\circ }$C in spring, 27.23±1.12 and 17.27±2.11${}^{\circ }$C in summer, 19.87±1.21 and 11.14±2.42${}^{\circ }$C in autumn, and 9.66±1.10 and 3.11±0.51${}^{\circ }$C in winter. Relative humidity percentages (%) were 57.12±2.55%, 67.54±2.11%, 54.23±2.12%, and 64.71±1.22% in the same seasons, respectively.

All animals were housed and managed according to the farm procedures of animal care in aa semi-open free-stall barn (10 bulls in a paddock, 12 m${}^{2}$ per animal) with straw as bedding. The fattening period was initiated after 2 weeks of adaptation. All animals were weighed monthly by a precision scale (100 g sensitivity) and were fed ad libitum with the same diets including grower and finisher rations, which contained corn, potato and tomato pomace silage, barley straw, barley butter, pasta, corn, corn gluten meal, corn bran, sugar-beet pulp, soybean meal, sunflower meal, vitamin and mineral premix, limestone, and salt. The grower ration contained 13.80 % of crude protein and 10.20 MJ kg${}^{-1}$ of metabolizable energy on a dry matter basis and the finisher ration contained 10.30 % of crude protein and 11.50 MJ kg${}^{-1}$ of energy on a dry matter basis. All animals were provided with the same feeding practices throughout the entire experimental period.

At the end of the finishing period, the animals were slaughtered at a commercial slaughter facility according to standard practices. Mean slaughter age was 487.30±2.95 days. After 1–2 h of transportation and with no access to feed (water was available) for approximately 24 h after arrival, final weights (FW) were recorded immediately before slaughter.

In order to evaluate the data for fattening performance, the variables FW, fattening period (FP), and average daily weight gain (ADWG) for each animal were determined. Dry matter intake (DMI) was measured daily and the ADWG was calculated for the interval between the two weight measurements. The feed conversion rate (FCR) was calculated, dividing the total amount of food consumed (based on DMI) between the weighing days to the total weight gain (Mundan et al., 2012). In this study, apart from the general determination of the FP, DMI, FCR, and ADWG, each trait was evaluated based on the fattening periods between five selected target body weights (W1 = 100 kg, W2 = 200 kg, W3 = 300 kg, W4 = 400 kg, W5 = 450 kg) and days to reach (DTR) these targets were compared. In this respect, the impact of genotypes on each fattening performance trait based on different periods (W1–W2, W2–W3, W3–W4, and W4–W5) was also evaluated.

### 2.2 Candidate gene marker selection

The markers were selected from different sources as follows: (a) SNPs from genes that belonged to QTL mapping studies (http://www.animalgenome.org/QTLdb/cattle.html, last access: 18 January 2019) with respect to fattening performance, lipid metabolism, and appetite regulation; (b) SNPs in genes previously associated with meat production and quality or milk quality traits (which are potential molecular markers for both meat and milk quality and growth); (c) Databases such as dbSNP (http://www.ncbi.nlm.nih.gov/projects/SNP/, last access: 18 January 2019) and Ensembl (http://www.ensembl.org, last access: 18 January 2019) were used for confirmation and information of each marker.

### 2.3 Genomic DNA isolation and genotyping

Genomic DNA was purified from 4 mL blood samples obtained from the vena jugularis of each bull according to previously described phenol–chloroform method (Green and Sambrook, 2012). The concentration range (ng µL${}^{-1}$) and the ratio 260/280 were measured with a spectrophotometer (NanoDrop 2000c, Thermo Scientific, Wilmington, DE, USA) to evaluate DNA amount and purity, respectively.

**Table 1:** Primer sequences (from 5${}^{\prime }$ to 3${}^{\prime }$), PCR conditions, and restriction enzymes used for genotyping the polymorphisms in the current study.

Marker name${}^{*}$	Allele	Amplicon	Primers (5${}^{\prime }$ to 3${}^{\prime }$)	PCR conditions	Restriction	Reference
		(bp)			enzyme	
*LEP* A80V	C/T	458	F: 5${}^{\prime }$ GGGAAGGGCAGAAAGATAG 3${}^{\prime }$	94 ${}^{\circ }$C 2${}^{\prime }$ (94 ${}^{\circ }$C 30 s, 57 ${}^{\circ }$C 1${}^{\prime }$, 72 ${}^{\circ }$C 30 s) 35 cycles, 72 ${}^{\circ }$C 15${}^{\prime }$	*Hph*I	Oztabak et al. (2010)
			R: 5${}^{\prime }$CCAAGCTCTCCAAGCTCTC3${}^{\prime }$			
*FABP4*g.3691G > A	G/A	565	F: 5${}^{\prime }$ ACCCCTATGATGCTATTCCACA 3${}^{\prime }$	94 ${}^{\circ }$C 4${}^{\prime }$ (94 ${}^{\circ }$C 1${}^{\prime }$, 60 ${}^{\circ }$C 1${}^{\prime }$, 72 ${}^{\circ }$C 1.5${}^{\prime }$) 35 cycles, 72 ${}^{\circ }$C 5${}^{\prime }$	*Nla*III	Shin et al. (2012)
			R: 5${}^{\prime }$ ATACGGTTCACATTGAGAGGGA 3${}^{\prime }$			
*FABP4* g.2834C > G	C/G	590	F: 5${}^{\prime }$ GCTGCTCTCTCATGGTTAAGATGG 3${}^{\prime }$	94 ${}^{\circ }$C 4${}^{\prime }$ (94 ${}^{\circ }$C 1${}^{\prime }$, 60 ${}^{\circ }$C 1${}^{\prime }$, 72 ${}^{\circ }$C 1.5${}^{\prime }$) 35 cycles, 72 ${}^{\circ }$C 5${}^{\prime }$	*Hpy*188I	Shin et al. (2012)
			R: 5${}^{\prime }$ CCTTGACTTTCCTGTCATCTGG 3${}^{\prime }$			
*FABP4* g.3533T > A	T/A	555	F: 5${}^{\prime }$ ACTGCTGCCTATAGCAAACCAT 3${}^{\prime }$	94 ${}^{\circ }$C 5${}^{\prime }$ (94 ${}^{\circ }$C 1${}^{\prime }$, 60 ${}^{\circ }$C 1${}^{\prime }$, 72 ${}^{\circ }$C 1${}^{\prime }$) 40 cycles, 72 ${}^{\circ }$C 10${}^{\prime }$	*Csp*6I	Shin et al. (2012)
			R: 5${}^{\prime }$ TACGATGCTCTGTGGGGATAAT 3${}^{\prime }$			
*DGAT1K232A*	K/A	411	F: 5${}^{\prime }$ GCACCATCCTCTTCCTCAAG 3${}^{\prime }$	${}^{*}$94 ${}^{\circ }$C 4${}^{\prime }$, 10 cycles (94 ${}^{\circ }$C 60 s, 66 ${}^{\circ }$C 60 s; -1 ${}^{\circ }$C per cycle,	*Cfr*I	Lacorte et al. (2006)
			R: 5${}^{\prime }$ GGAAGCGCTTTCGGATG 3${}^{\prime }$	72 ${}^{\circ }$C 60 s), 25 cycles (94 ${}^{\circ }$C 60 s, 56 ${}^{\circ }$C 120 s, 72 ${}^{\circ }$C 60 s),		
				72 ${}^{\circ }$C 15${}^{\prime }$		
*TG*C422T	C/T	545	F: 5${}^{\prime }$ GGGGATGACTACGAGTATGACTG 3${}^{\prime }$	94 ${}^{\circ }$C 5${}^{\prime }$ (94 ${}^{\circ }$C 1${}^{\prime }$, 55 ${}^{\circ }$C 1${}^{\prime }$, 72 ${}^{\circ }$C 1${}^{\prime }$) 35 cycles, 72 ${}^{\circ }$C 7${}^{\prime }$	*Mfl*I	Shin and Chung (2007b)
			R: 5${}^{\prime }$ GTGAAAATCTTGTGGAGGCTGTA 3${}^{\prime }$			
*IGF1*C472T	C/T	249	F: 5 ${}^{\prime }$ATTACAAAGCTGCCTGCCCC 3${}^{\prime }$	R: 94 ${}^{\circ }$C 5${}^{\prime }$ (94 ${}^{\circ }$C 1${}^{\prime }$, 64 ${}^{\circ }$C 1${}^{\prime }$, 72 ${}^{\circ }$C 1${}^{\prime }$) 31 cycles, 72 ${}^{\circ }$C 5${}^{\prime }$	*SnaB*I	Siadkowska et al. (2006)
			5${}^{\prime }$ ACCTTACCCGTATGAAAGGAATATACGT 3${}^{\prime }$			
*IGF1R*G404T	A/B	625	F: 5${}^{\prime }$ CCCAATGGATTGATCCTCATGT 3${}^{\prime }$	94 ${}^{\circ }$C 5${}^{\prime }$ (94 ${}^{\circ }$C 1${}^{\prime }$, 56 ${}^{\circ }$C 1${}^{\prime }$, 72 ${}^{\circ }$C 1${}^{\prime }$) 35 cycles, 72 ${}^{\circ }$C 5${}^{\prime }$	*Taq*I	Curi et al. (2005a)
			R: 5${}^{\prime }$ GCTGTGTAGTTCCCTGGGTT3 ${}^{\prime }$			
*MYF5*g.1911A > G	A/G	445	F: 5${}^{\prime }$ ACAGCGTCTACTGTCCTGATG 3${}^{\prime }$	94 ${}^{\circ }$C 4${}^{\prime }$ (94 ${}^{\circ }$C 30 s, 58 ${}^{\circ }$C 60 s, 72 ${}^{\circ }$C 60 s) 38 cycles, 72 ${}^{\circ }$C 4${}^{\prime }$	*Taq*I	Kisacova et al. (2009)
			R: 5${}^{\prime }$ CGTGGTATATACTAAGGACAC 3${}^{\prime }$			
*LGB*T5261C	A/B	247	F: 5${}^{\prime }$ TGTGCTGGACACCGACTACAAAAAG 3${}^{\prime }$	95 ${}^{\circ }$C 5${}^{\prime }$ (94 ${}^{\circ }$C 1.5${}^{\prime }$, 58 ${}^{\circ }$C 1${}^{\prime }$, 72 ${}^{\circ }$C 2${}^{\prime }$) 30 cycles, 72 ${}^{\circ }$C 10${}^{\prime }$	*Hae*III	Curi et al. (2005b)
			R: 5${}^{\prime }$ GCTCCCGGTATATGACCACCCTCT 3${}^{\prime }$			
*CAPN1*G316A	C/G	415	F: 5${}^{\prime }$ GACTGGGGTCTCTGGACTT 3${}^{\prime }$	95 ${}^{\circ }$C 5${}^{\prime }$ (95 ${}^{\circ }$C 45 s, 63 ${}^{\circ }$C 45 s, 72 ${}^{\circ }$C 45 s) 35 cycles, 72 ${}^{\circ }$C 5${}^{\prime }$	*Btg*I	Lisa and Di Stasio (2009)
			R: 5${}^{\prime }$ GGAACCTCTGGCTCTTGA 3${}^{\prime }$			
*CAPN1*V530I	A/G	787	F: 5${}^{\prime }$ AGCGCAGGGACCCAGTGA 3${}^{\prime }$	95 ${}^{\circ }$C 5${}^{\prime }$ (95 ${}^{\circ }$C 1${}^{\prime }$, 63 ${}^{\circ }$C 1${}^{\prime }$, 72 ${}^{\circ }$C 1${}^{\prime }$) 35 cycles, 72 ${}^{\circ }$C 5${}^{\prime }$	*Ava*II	Soria et al. (2010)
			R: 5${}^{\prime }$ TCCCCTGCCAGTTGTCTGAAG 3${}^{\prime }$			
*CAST* S20T	G/C	624	F: 5${}^{\prime }$ TGGGGCCCAATGACGCCATCGATG 3${}^{\prime }$	94 ${}^{\circ }$C 5${}^{\prime }$ (94 ${}^{\circ }$C 30 s, 62 ${}^{\circ }$C 45 s, 72 ${}^{\circ }$C 45 s) 32 cycles, 72 ${}^{\circ }$C 5${}^{\prime }$	*Alu*I	Juszczuk-Kubiak et al. (2004)
			R: 5${}^{\prime }$ GGTGGAGCAGCACTTCTGATCACC 3${}^{\prime }$			
*GHR* S555G	G/A	342	F: 5${}^{\prime }$ GCTAACTTCATCGTGGACAAC 3${}^{\prime }$	95 ${}^{\circ }$C 5${}^{\prime }$ (94 ${}^{\circ }$C 45 s, 53 ${}^{\circ }$C 30 s, 72 ${}^{\circ }$C 50 s) 35 cycles, 72 ${}^{\circ }$C 5${}^{\prime }$	*Alu*I	Di Stasio et al. (2005)
			R: 5${}^{\prime }$ CTATGGCATGATTTTGTTCAG 3${}^{\prime }$			
*OLR1* g.8232C > A	A/C	143	F: 5${}^{\prime }$ TCCCTAACTTGTTCCAAGTCCT 3${}^{\prime }$	94 ${}^{\circ }$C 5${}^{\prime }$ (94 ${}^{\circ }$C 30 s, 62 ${}^{\circ }$C 30 s, 72 ${}^{\circ }$C 40 s) 30 cycles, 72 ${}^{\circ }$C 5${}^{\prime }$	*Pst*I	Komisarek and Dorynek (2009)
			R: 5${}^{\prime }$ CTCTACAATGCCTAGAAGAAAGC 3${}^{\prime }$			

*LEP* – leptin; *FABP4* – fatty acid binding protein 4; *DGAT1* – diacylglycerol acyltransferase 1; *TG* – thyroglobulin; *IGF1* – insulin-like growth factor 1; *IGF1R* – insulin-like growth factor receptor 1; *MYF5* – myogenic factor 5; *LGB* – beta-lactoglobulin; *CAPN1* – micromolar calcium-activated neutral protease 1; *CAST* – calpastatin; *GHR* – growth hormone receptor; *OLR1* – oxidized low-density lipoprotein receptor. ${}^{*}$ Marker names were selected according to genomic regions or amino acid alterations.

Genotyping of the SNPs in the selected candidate genes was performed by PCR-RFLP (polymerase chain reaction – restriction fragment length polymorphism). Primer sequences and PCR conditions for amplification are shown in Table 1. The primer sequences were checked for specificity by conducting BLAST searches of the NCBI GenBank database. PCR reaction mixtures consisted of 1 µL (0.025 µM) of forward and reverse primers, 1 µL of dNTPs (2.5 mM), 5 µL of ${\mathrm{MgSO}}_{4}$, 2.5 units of Taq DNA polymerase (Biomatik, Cambridge, Canada), 5 µL 10 × reaction buffer (Fisher Biotech, Pittsburgh, PA), 3 µL of purified DNA, and 33.5 µL autoclaved Milli-Q water, obtained from a water purification system (Millipore, Bedford, MA, USA), to make a volume up to 50 µL. The DNA amplification reactions were performed in two thermal cyclers (Palm Cycler GC1-96, Corbett Research, Australia, and MyGenie 96 thermal block, Bioneer Corporation, South Korea). Amplification products were observed on a 2 % agarose gel (migration for 1 h at 100 V) using 5 µL of PCR product and 2 µL of loading buffer. Following PCR process, 15 µL of the amplified product with each SNP was digested with 15 units of the corresponding restriction enzyme (shown in Table 1). Restriction enzymes were purchased from MBI Fermentas (Canada) or Biomatik (Cambridge, Canada). These reactions were incubated at 37 ${}^{\circ }$C for 16 h. The samples were applied into the 3 % agarose gel (Sigma-Aldrich, Steinheim, Germany) with 1 × TBE buffer for electrophoretic separation. Ethidium bromide (1 µg mL${}^{-1}$) was used as an intercalated reagent and electrophoresis ran at a maximum voltage of 90 V per 1 h. DNA fragments were visualized with UV transillumination (DNR-Minilumi, DNR Bio-Imaging Systems, Israel). To determine the size of fragments we applied a DNA ladder (100–1000 bp, Biomatik).

### 2.4 Statistical analysis

The allelic and genotypic distribution presented in this paper were calculated for all SNPs by the standard procedure (Falconer and Mackay, 1996). The Hardy–Weinberg equilibrium (HWE) was tested for all alleles by using the POPGENE software v1.32 (Yeh et al., 2000). The population genetic indexes including gene homozygosity (Ho), gene heterozygosity (He), effective allele numbers (Ne), and polymorphism information content (PIC) were calculated, on the basis of allele frequencies, by using the following formulas as described by Botstein et al. (1980):

1Ho=∑i=1nPi2,2He=1-∑i=1nPi2,3Ne=1/∑i=1nPi2,4PIC=1-∑i=1nPi2-∑i=1n-1∑j=i+1n2Pi2Pj2,

where $P_{i}$ is the frequency of the ith allele and n is the number of alleles.

Statistical analyses were performed on records of fattening performance traits, including FW, DMI, FCR, and ADWG in purebred Holstein bulls (n=296). In order to evaluate the individual gene effects and interactions on different fattening periods, five target body weights (W1 = 100 kg, W2 = 200 kg, W3 = 300 kg, W4 = 400 kg, W5 = 450 kg) were selected. The proportionate adjustment of two weights (if necessary), depending upon their distance from the standard age, was estimated by linear interpolation method from the nearest weigh days.

The Minitab software (Minitab, Pennsylvania, USA, v17.1.0) was used to analyze the relationship between the individual or combined genotypes and performance traits in cattle. All analyses were done in two steps: initially, markers were evaluated using the significance of genotype effects for each trait; and afterwards, the interactions between the mentioned loci (two- and three-way interactions) were added to the model and tested for significance. For the association studies, the traits of interest were analyzed using the general linear model (GLM) procedure and an appropriate statistical model was selected according to coefficient of determination ($R^{2}$) to compare the explanatory power of models with different numbers of predictors. The statistical model included effects of age (days) and season (spring, summer, autumn, and winter) at the corresponding target weights (as mentioned above) and genotype. Age (days) was added to the model as a covariate, while season was added as a fixed effect. The mixed model selected was

5 $$Y_{ijklm}=\mu +\beta A_{i}+S_{j}+G_{k}+I_{l}+e_{ijklm},$$

where $Y_{ijklm}$ is the phenotypic observations; μ is the overall mean; $\beta A_{i}$ is the regression effect of age; $S_{j}$ is the fixed effect of season; $G_{k}$ is the fixed effect of genotypes; $I_{l}$ is the fixed effect of the genotypic interactions; and $e_{ijklm}$ is random error.

The sire effect was not included in the model since the number of genotyped bulls that were progenies of the same sire was very small, as indicated by Curi et al. (2006). In interaction analyses, genotypes with very low frequency were not included in the analysis in order to avoid unreliable results or confounding the influence of genotype effects on traits of interest. Taken together, the least square mean (LSM) estimates with standard errors (SE) for genotypes and fattening performance traits were used. For all statistical comparisons, a probability level of P<0.05 was accepted as statistically significant. When significant associations were identified, mean values were submitted for post hoc comparison by the Tukey test.

Additive effects and dominance deviation were also calculated using a reparameterized model as demonstrated by Falconer and Mackay (1996). In this respect, the additive effect was estimated as the difference between the means of two homozygous divided by two (a), whereas the dominance effect was calculated as the deviation of the heterozygote from the mean of the two homozygotes (d). The degree of dominance was evaluated according to d/a.

**Table 2:** Allele and genotype frequencies for the 15 markers in the total sample of animals (n=296).

Marker	Allele frequency			Genotype frequency${}^{*}$		
*LEP*	C	T		CC	CT	TT
	0.22	0.78		0.145 (43)	0.152 (45)	0.703 (208)
*FABP4* 3691	A	G		AA	GA	GG
	0.25	0.75		0.038 (11)	0.422 (125)	0.540 (160)
*FABP4* 2834	C	G		CC	GC	GG
	0.42	0.58		0.108 (32)	0.625 (185)	0.267 (79)
*FABP4* 3533	A	T		AA	TA	TT
	0.45	0.55		0.141 (42)	0.608 (180)	0.251 (74)
*DGAT1*	A	K		AA	KA	KK
	0.49	0.51		0.148 (44)	0.676 (200)	0.176 (52)
*TG*	C	T		CC	CT	TT
	0.88	0.12		0.804 (238)	0.155 (46)	0.040 (12)
*IGF1*	C	T		CC	CT	TT
	0.44	0.56		0.111 (33)	0.655 (194)	0.234 (69)
*IGF1R*	A	B		AA	AB	BB
	0.65	0.35		0.449 (133)	0.409 (121)	0.142 (42)
*MYF5*	A	G		AA	GA	GG
	0.28	0.72		0.034 (10)	0.483 (143)	0.483 (143)
*LGB*	A	B		AA	AB	BB
	0.44	0.56		0.122 (36)	0.645 (191)	0.233 (69)
*CAPN1* 316	C	G		CC	GC	GG
	0.29	0.71		0.061 (18)	0.462 (137)	0.477 (141)
*CAPN1* 530	A	G		AA	GA	GG
	0.17	0.83		0.034 (10)	0.273 (81)	0.693 (205)
*CAST*	C	G		CC	GC	GG
	0.57	0.43		0.304 (90)	0.527 (156)	0.169 (50)
*GHR*	A	G		AA	GA	GG
	0.78	0.22		0.662 (196)	0.229 (68)	0.109 (32)
*OLR1*	A	C		AA	AC	CC
	0.62	0.38		0.236 (70)	0.764 (226)	0 (0)

${}^{*}$ The number of animals per genotype is presented in parentheses.

## 3 Results

### 3.1 Allelic and genotypic distributions and population genetic indices

Table 2 shows the genotypic and allelic frequencies for the 15 markers studied in Holstein bulls. Two alleles and three genotypes in each SNP were observed except for the *OLR1* (CC genotype was not present). The minor allele frequencies (MAF) ranged from 0.12 to 0.49. With respect to the *TG* and *CAPN1* 530 polymorphisms, the frequencies of C (0.88) and G (0.83) alleles, respectively, were remarkably high in the current study. The frequency of heterozygous genotypes was quite high (≥0.65) in *DGAT1*, *IGF1*, *LGB*, and *OLR1* markers. Concerning *LEP*, *TG*, and *CAPN1* 530 markers, the number of animals with the reference homozygote genotype was rather high (>200 individuals) compared to heterozygotes and homozygotes for the variant allele in the population studied. It is worth noting that the frequency of heterozygous genotype was considerably low (<0.20) at the *LEP* and *TG* markers only.

**Table 3:** Genetic diversity at the selected genes in the total sample of animals (n=296).

Marker	$\chi^{2}$ (HWE)	P (HWE)	Ho	He	Ne	PIC
*LEP*	92.452	0.000${}^{***}$	0.6568	0.3432	1.5225	0.2843
*FABP4* 3691	5.098	0.023${}^{*}$	0.6249	0.3751	1.6000	0.3047
*FABP4* 2834	23.594	0.000${}^{***}$	0.5128	0.4872	1.9501	0.3685
*FABP4* 3533	15.740	0.001${}^{***}$	0.5050	0.4950	1.9802	0.3725
*DGAT1*	36.746	0.000${}^{***}$	0.5002	0.4998	1.9992	0.3749
*TG*	19.207	0.000${}^{***}$	0.7888	0.2112	1.2677	0.1889
*IGF1*	32.331	0.000${}^{***}$	0.5072	0.4928	1.9716	0.3714
*IGF1R*	2.791	0.094	0.5450	0.4550	1.8349	0.3515
*MYF5*	13.132	0.001${}^{***}$	0.5968	0.4032	1.6756	0.3219
*LGB*	27.858	0.000${}^{***}$	0.5072	0.4928	1.9716	0.3714
*CAPN1* 316	4.182	0.041${}^{*}$	0.5882	0.4118	1.7001	0.3270
*CAPN1* 530	0.323	0.569	0.7178	0.2822	1.3931	0.2424
*CAST*	1.606	0.205	0.5098	0.4902	1.9616	0.3701
*GHR*	33.621	0.000${}^{***}$	0.6568	0.3432	1.5225	0.2843
*OLR1*	112.861	0.000${}^{***}$	0.5288	0.4712	1.8911	0.3602

$\chi^{2}$ (HWE) – Hardy–Weinberg equilibrium $\chi^{2}$ value; Ho – gene homozygosity; He – gene heterozygosity; Ne – effective allele number; PIC – polymorphism information content. ${}^{*}$ P<0.05, ${}^{***}$ P<0.001: not consistent with equilibrium.

Hardy–Weinberg equilibrium (HWE) of SNPs is shown in Table 3. The distributions of genotypes were in agreement with HWE within *IGF1R*, *CAPN1* V530I, and *CAST* S20T, whereas deviations from HWE were observed for all remaining markers (P<0.05). The values of four population genetic indices (gene homozygosity, Ho; gene heterozygosity, He; effective allele number, Ne; and polymorphism information content, PIC) to evaluate the genetic diversity in the present Holstein population are also presented in Table 3. Ho was higher than 0.50 for all the markers studied. He values ranging from 0.2112 to 0.4998 were observed, whereas values of Ne approached 2.00 (ranging from 1.2677 to 1.9992) in the chi-square statistics. All values of PIC were above 0.10 and below 0.40 in the present study. The *TG* C422T and *CAPN1* V530I showed the low frequency of the alleles T (0.12) and A (0.17), respectively, resulting in low genetic variabilities in He, Ne, and PIC compared to the other SNPs showing relatively high values of population genetic indices. In this context, the highest He value (0.4998) was observed for the *DGAT1* marker. The results indicated that *FABP4* 2834, *FABP4* 3533, *DGAT1*, *IGF1*, *LGB*, and *CAST* markers were characterized by high values of Ne (>0.95). Moreover, same markers also exhibited relatively high values of PIC in this study.

**Table 4:** Summary of associations observed (significant associations are in bold).

	*LEP*	*FABP4*	*FABP4*	*FABP4*	*DGAT1*	*TG*	*IGF1*	*IGF1R*	*MYF5*	*LGB*	*CAPN1*	*CAPN1*	*CAST*	*GHR*	*OLR1*
		3691	2834	3533							316	530			
DTRW1	0.728	0.394	0.728	0.253	0.430	0.440	0.097	0.987	0.192	0.220	0.210	0.554	0.814	0.071	0.121
DTRW2	**0.001**	0.272	0.711	0.185	0.147	0.619	0.110	0.720	0.292	0.121	0.577	0.470	0.910	0.182	0.087
DTRW3	**0.008**	**0.037**	0.502	0.283	0.697	0.986	0.386	0.554	0.548	0.141	0.348	0.365	0.797	**0.034**	0.898
DTRW4	0.552	0.073	0.962	0.339	0.467	0.646	0.554	0.229	0.444	0.373	**0.005**	0.874	0.141	0.912	0.563
DTRW5	**0.006**	0.091	0.189	0.083	0.249	0.306	0.592	0.397	0.218	0.989	0.256	0.502	0.952	0.767	0.513
W1–W2 FP	0.374	0.267	0.298	0.161	0.338	0.891	0.153	0.256	0.335	0.647	0.420	0.909	0.963	0.557	0.534
W2–W3 FP	0.256	0.407	**0.015**	0.884	0.158	0.887	0.855	0.983	0.661	**0.047**	0.868	0.999	0.478	**0.006**	0.993
W3–W4 FP	0.944	0.957	0.370	0.208	0.594	0.409	0.425	0.151	0.203	0.317	0.588	0.815	0.373	0.303	0.061
W4–W5 FP	0.871	0.601	0.474	0.100	0.611	0.905	0.085	0.917	0.133	0.617	0.113	0.651	0.295	**0.019**	0.300
FW	0.966	0.211	0.144	0.192	0.745	0.106	0.494	0.291	0.986	0.262	**0.003**	0.554	0.204	0.123	0.252
W1–W2 DMI	0.269	0.727	0.594	0.369	0.308	0.748	0.668	0.136	0.864	0.658	0.614	0.575	0.273	0.577	0.913
W2–W3 DMI	0.599	0.105	0.052	0.792	0.173	0.203	0.843	0.224	0.224	0.104	0.832	0.724	**0.042**	**0.006**	0.167
W3–W4 DMI	0.758	0.782	0.492	0.136	0.558	0.747	0.399	0.709	0.349	0.786	0.869	0.807	0.980	0.259	0.056
W4–W5 DMI	0.738	0.666	0.283	0.134	0.517	0.671	0.168	0.560	0.255	0.812	0.166	0.912	0.241	0.167	0.784
TDMI	**0.018**	0.067	0.200	0.173	0.565	0.388	**0.020**	0.403	0.531	0.100	0.192	0.790	0.986	**0.000**	0.103
W1–W2 DDMI	0.518	0.554	0.765	**0.004**	0.967	0.818	0.126	0.056	0.370	0.298	0.362	0.475	0.718	0.668	0.344
W2–W3 DDMI	0.555	0.177	0.552	0.710	0.515	0.368	0.917	0.289	0.591	0.513	0.918	0.518	0.183	0.901	0.165
W3–W4 DDMI	0.822	0.794	0.060	0.772	0.647	0.363	0.097	0.631	0.146	0.123	0.057	0.326	0.214	0.751	0.841
W4–W5 DDMI	0.623	0.846	0.994	0.345	0.854	0.894	0.263	0.695	**0.034**	0.408	0.470	0.112	0.555	**0.040**	0.058
TDDMI	**0.000**	0.325	0.839	**0.011**	0.970	0.353	**0.020**	0.372	0.231	**0.012**	0.058	0.838	0.938	0.387	0.088
W1–W2 FCR	0.264	0.691	0.566	0.456	0.275	0.657	0.662	0.136	0.801	0.703	0.639	0.519	0.208	0.682	0.749
W2–W3 FCR	0.596	0.162	0.052	0.729	0.242	0.165	0.872	0.224	0.290	0.088	0.840	0.732	**0.023**	**0.004**	0.260
W3–W4 FCR	0.733	0.814	0.661	0.160	0.578	0.663	0.404	0.058	0.215	0.731	0.784	0.833	0.993	0.182	**0.024**
W4–W5 FCR	0.659	0.299	0.338	**0.031**	0.534	0.058	0.196	0.167	0.555	0.474	0.161	0.695	0.950	0.779	0.581
TFCR	**0.048**	0.811	0.061	0.669	0.732	0.215	**0.004**	0.156	0.921	0.761	**0.001**	0.636	0.279	0.056	0.064
W1–W2 ADWG	0.503	0.351	0.243	0.088	0.707	0.822	0.163	0.113	0.666	0.754	0.373	0.926	0.591	0.292	0.379
W2–W3 ADWG	0.218	0.781	**0.019**	0.866	0.515	0.754	0.947	0.892	0.632	**0.036**	0.921	0.971	0.267	**0.009**	0.782
W3–W4 ADWG	0.637	0.994	0.885	0.347	0.776	0.273	0.100	0.084	0.094	0.356	0.546	0.717	0.799	0.330	0.119
W4–W5 ADWG	0.857	0.772	0.354	**0.019**	0.631	0.570	0.057	0.574	0.099	0.398	0.104	0.970	0.411	**0.047**	0.419
TADWG	0.998	0.211	0.172	0.230	0.915	0.081	0.200	0.203	0.899	0.305	**0.007**	0.397	0.216	0.104	0.504

W1 – 100 kg, W2 – 200 kg, W3 – 300 kg, W4 – 400 kg, W5 – 450 kg body weight; DTR – days to reach; FP – fattening period; FW – final weight; DMI – dry matter intake; TDMI – total dry matter intake throughout the entire experimental period; DDMI – daily dry matter intake; TDDMI – total daily dry matter intake throughout the entire experimental period; FCR – feed conversion rate; TFCR – total feed conversion rate throughout the entire experimental period; ADWG – average daily weight gain; TADG – total average daily weight gain throughout the entire experimental period.

**Table 5:** Effects of selected gene polymorphisms on the days to reach target body weights, corresponding fattening periods, and final weight.

Marker	DTRW1	DTRW2	DTRW3	DTRW4	DTRW5	W1–W2 FP	W2–W3 FP	W3–W4 FP	W4–W5 FP	FW
	(days)	(days)	(days)	(days)	(days)	(days)	(days)	(days)	(days)	(kg)
*LEP*										
CC	106.26±5.61	$231.47\pm 7.52^{a}$	$328.73\pm 11.29^{a}$	465.20±25.30	$499.40\pm 14.02^{a}$	89.80±4.52	95.17±4.32	83.10±10.20	82.40±6.51	433.21±14.84
CT	105.91±5.36	$220.37\pm 7.65^{\mathrm{ab}}$	$329.50\pm 11.81^{a}$	454.90±27.00	$494.26\pm 14.69^{\mathrm{ab}}$	87.44±4.23	100.01±4.57	83.31±10.80	18.68±6.96	431.74±15.00
TT	104.04±4.61	$214.13\pm 6.64^{b}$	$314.10\pm 10.14^{b}$	450.30±21.70	$477.59\pm 12.85^{b}$	90.58±3.69	97.31±3.86	84.66±8.62	83.43±5.96	434.15±13.36
*FABP4* 3691										
AA	109.85±7.00	230.39±9.82	$348.11\pm 16.51^{a}$	469.81±32.43	492.62±19.01	88.80±5.67	101.65±6.38	84.40±13.01	77.38±9.66	419.30±19.30
GA	102.41±4.73	217.94±6.79	$312.91\pm 10.10^{b}$	434.75±23.92	483.21±12.72	90.90±3.79	96.06±3.88	82.67±9.48	85.40±5.75	443.45±13.36
GG	103.96±4.46	217.65±6.27	$311.23\pm 9.33^{b}$	446.01±20.71	495.42±12.01	88.13±3.54	94.78±3.55	83.92±8.28	84.74±5.51	436.36±12.35
*FABP4* 2834										
CC	105.03±5.68	221.21±8.26	326.02±13.01	453.53±30.12	483.31±15.52	88.48±4.59	$101.87\pm 4.96^{a}$	78.10±12.00	84.13±7.57	439.91±16.24
GC	104.55±4.66	223.72±6.53	326.21±9.97	457.90±21.95	489.12±12.61	91.11±3.77	$97.48\pm 3.87^{\mathrm{ab}}$	88.19±8.75	80.19±5.94	435.74±12.86
GG	106.64±4.92	221.05±6.89	320.01±10.31	459.10±22.44	498.72±13.22	88.23±3.88	$93.13\pm 3.96^{b}$	84.73±9.03	83.20±6.18	423.54±13.40
*FABP4* 3533										
AA	105.17±5.49	218.09±7.64	318.70±11.81	454.80±26.22	497.45±14.40	90.35±4.26	96.80±4.54	83.21±10.52	86.73±6.82	427.45±15.04
TA	103.47±4.69	221.28±6.67	323.60±10.21	448.31±22.01	482.24±12.70	90.61±3.80	97.50±3.92	88.33±8.74	78.92±5.87	440.51±12.93
TT	107.58±5.13	226.60±7.27	330.01±11.11	467.33±25.21	491.62±14.00	86.86±4.16	98.18±4.29	79.51±10.20	81.86±6.66	431.13±14.36
*DGAT1*										
AA	105.88±5.47	223.57±7.83	322.84±12.12	463.51±29.21	492.34±14.66	88.58±4.43	97.24±4.62	79.1±11.70	81.46±6.86	428.92±15.37
KA	106.95±4.64	225.08±6.52	326.82±9.90	461.30±21.22	495.53±12.64	88.01±3.75	95.47±3.86	86.21±8.51	84.35±5.92	435.40±12.88
KK	103.38±5.10	217.32±7.24	322.62±11.12	445.70±23.80	483.43±13.96	91.23±4.02	99.77±4.28	85.68±9.50	81.70±6.71	434.75±14.36
*TG*										
CC	107.97±4.45	218.49±6.18	324.93±9.33	458.30±20.21	485.20±11.47	89.81±3.54	96.58±3.64	84.86±8.11	82.79±5.30	447.40±12.19
CT	104.67±4.92	219.77±6.97	324.61±10.72	446.61±24.20	478.30±13.00	90.29±3.98	97.30±4.12	90.24±9.71	81.31±6.20	444.65±13.70
TT	103.57±7.42	227.70±9.01	322.71±16.23	465.60±35.91	507.62±21.92	87.72±5.96	98.60±6.12	75.90±14.20	83.40±10.20	407.00±21.70
*IGF1*										
CC	102.94±5.89	227.76±8.13	330.10±12.51	467.51±27.32	491.52±15.30	92.17±4.69	98.18±4.87	78.51±11.00	79.01±7.18	426.72±15.96
CT	108.88±4.60	221.66±6.74	323.51±10.32	455.00±23.01	493.21±13.10	86.94±3.75	97.65±3.99	86.57±9.24	80.86±6.27	433.33±13.25
TT	104.40±4.88	216.55±6.89	318.77±10.52	447.91±23.71	486.42±13.01	88.71±3.89	96.65±4.02	85.98±9.41	87.64±6.21	439.16±13.57
*IGF1R*										
AA	109.83±6.54	214.92±8.42	320.60±11.20	469.20±24.11	500.70±17.01	89.44±3.89	97.37±4.09	94.10±10.80	82.07±6.35	430.11±13.50
AB	110.05±6.53	216.26±8.61	319.20±11.40	453.60±24.80	491.60±17.30	90.57±3.98	97.72±4.20	84.91±10.81	83.10±6.32	432.40±13.80
BB	109.52±7.19	211.67±9.38	312.71±12.60	447.00±26.30	485.40±18.51	86.32±4.31	97.48±4.52	87.30±11.31	81.85±6.78	444.11±15.00

**Table 5:** Continued.

Marker	DTRW1	DTRW2	DTRW3	DTRW4	DTRW5	W1–W2 FP	W2–W3 FP	W3–W4 FP	W4–W5 FP	FW
	(days)	(days)	(days)	(days)	(days)	(days)	(days)	(days)	(days)	(kg)
*MYF5*										
AA	110.33±6.81	227.60±9.89	322.86±14.72	449.20±31.31	488.91±19.21	93.28±5.42	99.41±5.59	84.41±12.40	90.46±8.59	432.35±19.64
GA	104.29±4.67	220.91±6.53	327.11±10.10	466.81±22.90	495.85±12.24	86.98±3.70	97.09±3.94	86.98±9.23	77.45±5.82	432.91±12.64
GG	101.60±4.74	217.46±6.49	322.32±10.01	454.50±22.50	486.56±12.24	87.56±3.68	95.98±3.89	79.61±8.98	79.60±6.01	433.80±12.63
*LGB*										
AA	101.65±5.53	219.24±7.76	333.18±11.91	472.21±27.22	491.31±15.10	90.84±4.36	$93.22\pm 4.62^{b}$	77.0±10.90	84.38±7.17	425.41±15.34
AB	107.66±4.71	226.38±6.76	320.36±10.37	449.00±23.00	490.02±12.72	88.94±3.85	$99.14\pm 3.97^{a}$	86.44±9.17	82.79±5.97	432.44±13.26
BB	106.91±5.11	220.36±7.19	318.94±10.90	449.30±24.40	489.90±13.50	88.04±4.07	$100.12\pm 4.15^{a}$	87.57±9.73	80.34±6.35	441.28±14.06
*CAPN1* 316										
CC	109.75±6.23	222.84±8.94	329.21±13.20	$474.54\pm 29.71^{a}$	485.32±12.50	86.41±5.02	96.89±5.07	78.50±12.00	76.65±8.05	4$05.34\pm 17.65^{b}$
GC	102.20±4.76	219.95±6.70	318.81±10.41	$428.14\pm 23.30^{b}$	482.96±12.70	91.09±3.78	98.20±3.97	85.34±9.15	87.28±6.03	$445.12\pm 13.71^{a}$
GG	104.27±4.60	223.18±6.50	324.21±0.01	$447.82\pm 22.40^{\mathrm{ab}}$	502.91±17.20	90.32±3.71	97.40±3.89	87.16±9.02	83.58±5.95	$448.65\pm 12.78^{a}$
*CAPN1* 530										
AA	109.94±6.92	229.18±9.95	336.01±14.31	464.32±32.40	478.21±19.20	88.70±5.64	97.62±5.51	79.30±12.91	85.62±8.72	423.35±19.64
GA	103.23±5.02	218.45±7.01	317.81±11.01	454.70±23.90	482.55±13.30	89.96±3.98	97.41±4.21	85.75±9.59	79.80±6.37	440.60±13.72
GG	103.05±4.90	218.34±6.78	318.51±10.61	451.40±22.90	487.46±12.90	89.17±3.85	97.46±4.07	86.02±9.10	82.09±6.21	435.10±13.34
*CAST*										
CC	104.55±4.93	221.10±6.98	325.71±10.70	467.80±23.50	490.70±13.66	89.57±3.97	99.00±4.14	87.97±9.54	80.07±6.46	426.90±13.75
GC	106.13±4.86	222.66±6.89	322.34±10.60	463.50±23.50	491.41±13.15	89.04±3.89	97.42±4.07	81.50±9.46	81.91±6.14	430.21±13.54
GG	105.54±5.27	222.21±7.48	324.20±11.47	439.10±25.81	489.10±14.13	89.21±4.21	96.07±4.38	81.60±10.20	85.54±6.56	441.92±14.60
*GHR*										
AA	101.48±4.63	217.62±6.51	$315.24\pm 9.94^{b}$	456.81±21.51	487.75±12.50	87.97±3.69	$93.44\pm 3.79^{b}$	88.50±8.60	$82.87\pm 5.87^{\mathrm{ab}}$	440.65±12.87
GA	104.12±5.13	222.60±7.19	$328.30\pm 11.11^{a}$	460.70±24.01	489.11±14.12	90.12±4.12	$99.84\pm 4.27^{a}$	81.35±9.68	$88.67\pm 6.56^{a}$	426.03±14.12
GG	110.62±5.87	225.76±8.14	$328.74\pm 12.10^{a}$	452.90±28.90	494.34±15.01	89.74±4.65	$99.20\pm 4.74^{\mathrm{ab}}$	81.21±11.50	$75.98\pm 7.08^{b}$	432.52±15.77
*OLR1*										
AA	107.36±5.10	225.02±7.20	324.44±11.12	453.41±24.21	488.37±13.60	89.89±4.10	97.50±4.28	79.34±9.71	80.97±6.44	436.90±14.01
AC	103.45±4.71	218.96±6.72	323.75±10.31	460.20±23.20	492.40±13.05	88.66±3.78	97.49±3.97	88.02±9.29	84.04±6.13	429.17±13.22

W1 – 100 kg, W2 – 200 kg, W3 – 300 kg, W4 – 400 kg, W5 – 450 kg body weight; DTR – days to reach; FP – fattening period; FW – final weight. ${}^{a,b}$ Different superscripts within a column indicate significant difference.

**Table 6:** Effects of selected gene polymorphisms on dry matter intake.

Marker	W1–W2	W2–W3	W3–W4	W4–W5	TDMI	W1–W2	W2–W3	W3–W4	W4–W5	TDDMI
	DMI (kg)	DMI (kg)	DMI (kg)	DMI (kg)	(kg)	DDMI	DDMI	DDMI	DDMI	(kg day${}^{-1}$)
						(kg day${}^{-1}$)	(kg day${}^{-1}$)	(kg day${}^{-1}$)	(kg day${}^{-1}$)	
*LEP*										
CC	419.54±16.34	622.81±23.10	639.52±56.01	725.62±42.36	$4017\pm 119^{\mathrm{ab}}$	4.67±0.18	6.69±0.21	7.54±0.29	8.98±0.38	$8.49\pm 0.24^{a}$
CT	407.62±15.46	638.63±24.44	634.44±59.26	704.40±45.20	$4150\pm 121^{a}$	4.69±0.17	6.55±0.22	7.66±0.31	8.85±0.40	$8.02\pm 0.21^{\mathrm{ab}}$
TT	420.55±13.54	629.46±20.77	654.92±47.43	717.40±38.84	$3965\pm 103^{b}$	4.78±0.15	6.66±0.19	7.63±0.25	8.79±0.34	$7.78\pm 0.25^{b}$
*FABP4* 3691										
AA	420.75±20.50	641.26±34.13	661.45±71.50	687.82±62.71	3943±156	4.79±0.23	6.57±0.31	7.71±0.38	8.99±0.56	7.98±0.32
GA	415.53±13.84	634.90±20.82	628.00±52.10	732.90±37.52	4144±107	4.66±0.15	6.74±0.19	7.53±0.28	8.85±0.33	8.22±0.22
GG	411.45±13.01	614.75±19.01	639.40±45.51	726.70±35.72	4045±98	4.72±0.14	6.58±0.17	7.59±0.24	8.78±0.32	8.09±0.20
*FABP4* 2834										
CC	414.73±16.70	655.53±26.58	612.80±66.01	726.72±49.20	4111±131	4.75±0.19	6.61±0.24	7.64±0.35	8.87±0.44	8.04±0.27
GC	420.01±13.70	626.06±20.73	651.20±48.15	696.91±38.61	3981±103	4.69±0.15	6.59±0.19	7.46±0.26	8.88±0.34	8.11±0.21
GG	413.06±14.26	609.37±21.25	664.86±49.72	723.82±40.21	4040±108	4.74±0.15	6.70±0.19	7.75±0.26	8.87±0.36	8.14±0.22
*FABP4* 3533										
AA	417.44±15.60	626.72±24.35	629.21±57.60	742.80±44.42	3967±121	$4.63\pm 0.17^{b}$	6.62±0.22	7.56±0.31	8.75±0.39	$8.33\pm 0.25^{a}$
TA	410.32±13.81	634.82±21.00	674.90±48.11	694.90±38.12	4098±104	$4.64\pm 0.15^{b}$	6.68±0.19	7.59±0.26	9.02±0.34	$7.91\pm 0.21^{b}$
TT	419.95±15.14	629.30±23.00	624.60±56.01	709.72±43.35	4066±115	$4.89\pm 0.17^{a}$	6.60±0.21	7.68±0.29	8.85±0.38	$8.05\pm 0.24^{\mathrm{ab}}$
*DGAT1*										
AA	416.23±16.11	639.12±24.72	625.00±64.41	705.50±44.61	4085±124	4.72±0.18	6.73±0.22	7.72±0.34	8.85±0.39	8.09±0.26
KA	409.90±13.61	618.11±20.76	662.12±46.82	729.32±38.42	4047±103	4.71±0.15	6.61±0.19	7.60±0.25	8.83±0.34	8.08±0.21
KK	421.50±14.75	633.72±22.97	641.61±52.32	712.72±43.61	3999±113	4.74±0.16	6.57±0.21	7.51±0.28	8.94±0.39	8.12±0.23
*TG*										
CC	416.45±12.91	618.23±19.50	649.22±44.63	710.45±34.42	4025±96	4.75±0.14	6.54±0.18	7.59±0.24	8.79±0.31	8.26±0.19
CT	421.60±14.62	610.62±22.00	663.92±53.41	695.52±40.40	4112±110	4.78±0.16	6.49±0.19	7.42±0.28	8.82±0.36	8.13±0.23
TT	409.61±21.65	662.12±32.80	615.72±78.16	741.61±65.72	3993±175	4.64±0.24	6.86±0.29	7.82±0.41	9.01±0.58	7.90±0.36
*IGF1*										
CC	421.85±17.01	624.44±26.14	615.01±60.44	696.5±46.70	$4020\pm 128^{\mathrm{ab}}$	4.66±0.19	6.59±0.24	7.66±0.32	9.06±0.41	$8.02\pm 0.26^{\mathrm{ab}}$
CT	413.13±13.75	633.83±21.35	645.90±50.80	706.9±40.70	$3981\pm 108^{b}$	4.83±0.15	6.66±0.19	7.43±0.27	8.91±0.36	$7.97\pm 0.22^{b}$
TT	412.70±14.12	632.72±21.58	667.81±51.72	744.0±40.40	$4131\pm 106^{a}$	4.69±0.16	6.65±0.19	7.75±0.27	8.65±0.36	$8.29\pm 0.22^{a}$
*IGF1R*										
AA	419.00±14.22	624.40±21.70	642.80±60.60	710.80±41.30	4180±108	4.67±0.16	6.58±0.19	7.66±0.32	8.92±0.37	7.96±0.15
AB	407.80±14.40	641.60±22.30	661.80±51.20	724.20±40.80	4135±111	4.56±0.17	6.78±0.20	7.43±0.27	8.87±0.36	7.94±0.16
BB	422.41±15.72	627.4±24.00	675.61±52.60	700.00±44.00	4091±121	4.81±0.18	6.61±0.22	7.75±0.28	8.74±0.39	7.82±0.17

**Table 6:** Continued.

Marker	W1–W2	W2–W3	W3–W4	W4–W5	TDMI	W1–W2	W2–W3	W3–W4	W4–W5	TDDMI
	DMI (kg)	DMI (kg)	DMI (kg)	DMI (kg)	(kg)	DDMI	DDMI	DDMI	DDMI	(kg day${}^{-1}$)
						(kg day${}^{-1}$)	(kg day${}^{-1}$)	(kg day${}^{-1}$)	(kg day${}^{-1}$)	
*MYF5*										
AA	420.62±20.25	637.45±29.90	632.22±68.21	761.91±55.80	3988±158	4.56±0.22	6.56±0.27	7.33±0.36	$8.74\pm 0.49^{b}$	8.16±0.32
GA	414.50±13.57	634.44±21.13	664.41±50.72	695.30±37.92	4093±102	4.80±0.15	6.70±0.19	7.68±0.27	$9.13\pm 0.34^{a}$	7.99±0.21
GG	412.61±13.35	619.14±20.85	632.21±49.40	690.23±39.01	4050±102	4.81±0.15	6.63±0.19	7.82±0.26	$8.75\pm 0.35^{b}$	8.15±0.21
*LGB*										
AA	415.50±15.91	610.73±24.71	626.72±60.00	721.82±46.63	3998±123	4.71±0.18	6.73±0.22	7.87±0.32	8.69±0.41	$8.37\pm 0.25^{a}$
AB	412.82±14.05	638.00±21.24	646.71±50.40	718.70±38.82	4008±105	4.67±0.16	6.59±0.19	7.49±0.27	8.89±0.34	$7.92\pm 0.22^{b}$
BB	419.37±14.72	642.25±22.20	655.40±53.51	707.00±41.32	4125±113	4.79±0.16	6.57±0.20	7.47±0.28	9.03±0.37	$7.99\pm 0.23^{b}$
*CAPN1* 316										
CC	409.43±18.23	634.10±27.10	637.61±66.10	681.21±52.35	4164±142	4.79±0.20	6.66±0.25	7.99±0.35	9.04±0.46	8.15±0.29
GC	417.15±13.82	630.80±21.20	639.45±50.30	743.80±39.13	3989±105	4.65±0.15	6.63±0.19	7.46±0.27	8.72±0.35	7.95±0.22
GG	421.08±13.54	625.90±20.80	651.72±49.60	722.42±38.72	3978±103	4.73±0.15	6.61±0.19	7.39±0.26	8.86±0.34	8.19±0.21
*CAPN1* 530										
AA	424.22±20.53	643.00±29.50	630.44±70.91	702.52±56.53	4022±158	4.83±0.23	6.81±0.27	7.77±0.38	8.43±0.50	8.13±0.33
GA	414.21±14.50	622.00±22.50	657.43±52.72	724.33±41.54	4073±110	4.70±0.16	6.54±0.20	7.62±0.28	9.23±0.37	8.05±0.23
GG	409.30±13.91	625.80±21.80	640.93±50.00	720.70±40.40	4037±107	4.64±0.15	6.55±0.19	7.44±0.27	8.95±0.36	8.12±0.22
*CAST*										
CC	417.35±14.47	$638.62\pm 22.21^{a}$	646.55±52.50	696.10±42.00	4049±110	4.69±0.16	6.61±0.20	7.46±0.28	8.89±0.37	8.10±0.23
GC	421.10±15.54	$641.40\pm 21.87^{a}$	642.00±52.01	717.80±39.90	4041±109	4.70±0.16	6.74±0.19	7.69±0.28	8.96±0.35	8.07±0.22
GG	409.20±14.13	$610.84\pm 23.46^{b}$	640.33±56.30	733.50±42.71	4041±118	4.77±0.17	6.56±0.21	7.69±0.29	8.76±0.38	8.11±0.24
*GHR*										
AA	415.22±13.52	$607.85\pm 20.37^{b}$	652.72±47.35	720.52±38.14	$4213\pm 103^{a}$	4.74±0.15	6.65±0.18	7.56±0.25	$8.87\pm 0.34^{\mathrm{ab}}$	8.01±0.21
GA	421.80±15.01	$640.73\pm 22.95^{\mathrm{ab}}$	643.20±53.21	741.22±42.76	$4017\pm 114^{b}$	4.67±0.17	6.60±0.21	7.67±0.28	$8.55\pm 0.38^{b}$	8.04±0.23
GG	410.64±16.80	$642.30\pm 25.33^{a}$	612.91±63.24	685.81±46.06	$3902\pm 127^{b}$	4.76±0.19	6.65±0.23	7.60±0.34	$9.19\pm 0.41^{a}$	8.24±0.26
*OLR1*										
AA	415.51±14.94	622.52±22.90	617.42±53.40	713.21±41.90	3999±113	4.69±0.17	6.56±0.21	7.59±0.28	9.03±0.37	8.00±0.23
AC	416.37±13.85	638.10±21.30	638.41±51.12	718.42±39.80	4089±107	4.76±0.15	6.70±0.19	7.62±0.27	8.71±0.35	8.19±0.22

W1 – 100 kg, W2 – 200 kg, W3 – 300 kg, W4 – 400 kg, W5 – 450 kg body weight; DMI – dry matter intake; TDMI – total dry matter intake throughout the entire experimental period; DDMI – daily dry matter intake; TDDMI – total daily dry matter intake throughout the entire experimental period. ${}^{a,b}$ Different superscripts within a column indicate significant difference.

**Table 7:** Effects of selected gene polymorphisms on feed conversion rate and average daily gain.

Marker	W1–W2 FCR	W2–W3 FCR	W3–W4 FCR	W4–W5 FCR	TFCR	W1–W2	W2–W3	W3–W4	W4–W5	TADWG
	(kg kg${}^{-1}$)	(kg kg${}^{-1}$)	(kg kg${}^{-1}$)	(kg kg${}^{-1}$)	(kg kg${}^{-1}$)	ADWG	ADWG	ADWG	ADWG	(kg)
						(kg)	(kg)	(kg)	(kg)	
*LEP*										
CC	4.19±0.16	6.23±0.23	6.33±0.55	13.66±0.73	$9.63\pm 0.36^{a}$	1.16±0.05	1.09±0.05	1.17±0.08	1.24±0.11	0.89±0.03
CT	4.08±0.16	6.39±0.25	6.17±0.58	13.31±0.79	$9.64\pm 0.37^{a}$	1.19±0.05	1.03±0.05	1.19±0.08	1.25±0.11	0.90±0.03
TT	4.21±0.14	6.31±0.21	6.42±0.47	13.65±0.67	$9.27\pm 0.32^{b}$	1.16±0.04	1.06±0.04	1.15±0.06	1.22±0.09	0.89±0.04
*FABP4* 3691										
AA	4.20±0.21	6.43±0.34	6.46±0.69	12.57±1.12	9.62±0.47	1.18±0.06	1.04±0.07	1.17±0.09	1.29±0.16	0.87±0.04
GA	4.17±0.14	6.34±0.21	6.17±0.51	14.01±0.65	9.54±0.33	1.15±0.04	1.07±0.04	1.17±0.07	1.20±0.09	0.91±0.03
GG	4.12±0.13	6.16±0.19	6.28±0.45	14.02±0.61	9.38±0.29	1.18±0.04	1.08±0.04	1.17±0.06	1.20±0.09	0.89±0.03
*FABP4* 2834										
CC	4.17±0.17	6.54±0.27	6.09±0.65	13.72±0.85	9.47±0.39	1.19±0.05	$1.02\pm 0.06^{b}$	1.18±0.09	1.19±0.12	0.91±0.03
GC	4.20±0.14	6.28±0.22	6.35±0.47	13.24±0.67	9.26±0.31	1.15±0.04	$1.06\pm 0.04^{\mathrm{ab}}$	1.16±0.07	1.28±0.09	0.89±0.02
GG	4.12±0.14	6.12±0.21	6.48±0.49	13.65±0.70	9.65±0.33	1.18±0.05	$1.11\pm 0.04^{a}$	1.17±0.07	1.22±0.10	0.88±0.03
*FABP4* 3533										
AA	4.19±0.16	6.25±0.24	6.21±0.56	$13.85\pm 0.79^{a}$	9.38±0.36	1.14±0.05	1.06±0.05	1.18±0.08	$1.16\pm 0.11^{b}$	0.88±0.03
TA	4.11±0.14	6.36±0.21	6.59±0.47	$13.02\pm 0.67^{b}$	9.44±0.31	1.17±0.04	1.07±0.04	1.14±0.07	$1.31\pm 0.09^{a}$	0.91±0.02
TT	4.19±0.15	6.32±0.23	6.11±0.55	$13.73\pm 0.74^{\mathrm{ab}}$	9.57±0.35	1.20±0.05	1.06±0.05	1.19±0.07	$1.21\pm 0.10^{\mathrm{ab}}$	0.89±0.03
*DGAT1*										
AA	4.17±0.16	6.37±0.25	6.16±0.63	13.55±0.77	9.62±0.38	1.16±0.05	1.06±0.05	1.19±0.09	1.23±0.11	0.89±0.03
KA	4.10±0.14	6.20±0.21	6.49±0.46	13.75±0.68	9.51±0.31	1.18±0.04	1.08±0.04	1.16±0.06	1.21±0.09	0.90±0.02
KK	4.22±0.15	6.36±0.23	6.27±0.51	13.31±0.76	9.43±0.35	1.17±0.05	1.05±0.05	1.17±0.07	1.26±0.11	0.91±0.03
*TG*										
CC	4.18±0.13	6.19±0.19	6.34±0.44	13.22±0.61	9.24±0.29	1.18±0.04	1.07±0.04	1.17±0.06	1.25±0.09	0.92±0.03
CT	4.23±0.15	6.10±0.22	6.54±0.52	12.59±0.71	9.49±0.34	1.17±0.04	1.08±0.05	1.12±0.07	1.29±0.10	0.91±0.03
TT	4.08±0.22	6.65±0.33	6.03±0.76	14.80±1.10	9.84±0.53	1.15±0.07	1.04±0.07	1.23±0.11	1.15±0.17	0.84±0.04
*IGF1*										
CC	4.22±0.17	6.27±0.26	6.03±0.59	13.11±0.81	$9.63\pm 0.39^{\mathrm{ab}}$	1.14±0.05	1.07±0.05	1.23±0.08	1.31±0.12	0.88±0.03
CT	4.13±0.14	6.35±0.22	6.34±0.49	13.49±0.69	$9.72\pm 0.32^{a}$	1.19±0.04	1.06±0.04	1.13±0.07	1.25±0.10	0.89±0.03
TT	4.14±0.14	6.32±0.21	6.55±0.51	14.02±0.72	$9.21\pm 0.33^{b}$	1.17±0.04	1.06±0.04	1.16±0.07	1.14±0.10	0.91±0.02
*IGF1R*										
AA	4.19±0.14	6.24±0.22	6.64±0.51	13.37±0.71	9.68±0.33	1.15±0.04	1.07±0.04	1.13±0.07	1.26±0.10	0.88±0.03
AB	4.08±0.14	6.42±0.22	6.09±0.52	13.90±0.69	9.61±0.34	1.15±0.04	1.06±0.05	1.20±0.07	1.22±0.11	0.89±0.02
BB	4.22±0.16	6.27±0.24	6.19±0.55	13.34±0.76	9.27±0.37	1.21±0.05	1.06±0.05	1.18±0.08	1.24±0.11	0.91±0.03

**Table 7:** Continued.

Marker	W1–W2 FCR	W2–W3 FCR	W3–W4 FCR	W4–W5 FCR	TFCR	W1–W2	W2–W3	W3–W4	W4–W5	TADWG
	(kg kg${}^{-1}$)	(kg kg${}^{-1}$)	(kg kg${}^{-1}$)	(kg kg${}^{-1}$)	(kg kg${}^{-1}$)	ADWG	ADWG	ADWG	ADWG	(kg)
						(kg)	(kg)	(kg)	(kg)	
*MYF5*										
AA	4.21±0.20	6.40±0.30	6.12±0.67	14.07±0.98	9.47±0.48	1.14±0.06	1.04±0.06	1.16±0.093	1.12±0.14	0.88±0.04
GA	4.16±0.14	6.33±0.21	6.58±0.49	13.26±0.65	9.57±0.31	1.18±0.04	1.07±0.04	1.15±0.07	1.32±0.09	0.90±0.03
GG	4.13±0.13	6.19±0.21	6.21±0.48	13.28±0.68	9.52±0.31	1.19±0.04	1.08±0.04	1.21±0.07	1.25±0.09	0.90±0.03
*LGB*										
AA	4.16±0.16	6.11±0.25	6.13±0.59	13.87±0.79	9.61±0.37	1.16±0.05	$1.11\pm 0.05^{a}$	1.22±0.08	1.17±0.12	0.88±0.03
AB	4.14±0.14	6.39±0.22	6.34±0.49	13.47±0.68	9.45±0.32	1.17±0.04	$1.05\pm 0.04^{\mathrm{ab}}$	1.16±0.07	1.25±0.09	0.89±0.02
BB	4.19±0.15	6.44±0.22	6.45±0.52	13.27±0.72	9.51±0.34	1.18±0.05	$1.03\pm 0.05^{b}$	1.14±0.07	1.27±0.10	0.91±0.03
*CAPN1* 316										
CC	4.09±0.18	6.36±0.27	6.15±0.65	12.99±0.93	$10.27\pm 0.43^{a}$	1.21±0.06	1.05±0.06	1.22±0.09	1.31±0.13	$0.84\pm 0.04^{b}$
GC	4.19±0.14	6.31±0.21	6.33±0.49	14.02±0.67	$9.09\pm 0.32^{b}$	1.15±0.04	1.07±0.04	1.15±0.07	1.15±0.09	$0.92\pm 0.03^{a}$
GG	4.21±0.14	6.27±0.22	6.44±0.48	13.61±0.67	$9.03\pm 0.31^{b}$	1.15±0.04	1.07±0.04	1.15±0.07	1.23±0.09	$0.92\pm 0.03^{a}$
*CAPN1* 530										
AA	4.25±0.21	6.44±0.29	6.19±0.69	13.12±0.96	9.72±0.47	1.18±0.06	1.07±0.06	1.21±0.09	1.22±0.14	0.87±0.04
GA	4.15±0.15	6.23±0.23	6.44±0.52	13.84±0.75	9.31±0.33	1.16±0.04	1.06±0.05	1.16±0.07	1.25±0.10	0.91±0.03
GG	4.09±0.14	6.27±0.22	6.29±0.49	13.66±0.72	9.35±0.32	1.17±0.04	1.06±0.05	1.15±0.06	1.24±0.10	0.89±0.03
*CAST*										
CC	4.17±0.15	$6.41\pm 0.22^{\mathrm{ab}}$	6.31±0.51	13.47±0.73	9.60±0.33	1.16±0.04	1.04±0.05	1.16±0.07	1.25±0.11	0.88±0.03
GC	4.09±0.14	$6.43\pm 0.22^{a}$	6.29±0.51	13.56±0.69	9.51±0.33	1.18±0.04	1.06±0.04	1.18±0.07	1.26±0.10	0.89±0.03
GG	4.23±0.16	$6.10\pm 0.24^{b}$	6.32±0.55	13.58±0.74	9.27±0.35	1.17±0.05	1.08±0.05	1.18±0.08	1.19±0.11	0.91±0.03
*GHR*										
AA	4.16±0.14	$6.01\pm 0.20^{b}$	6.64±0.46	13.63±0.67	9.77±0.31	1.18±0.04	$1.11\pm 0.04^{a}$	1.14±0.07	$1.22\pm 0.09^{\mathrm{ab}}$	0.91±0.03
GA	4.21±0.15	$6.43\pm 0.23^{a}$	6.32±0.52	13.64±0.75	9.59±0.35	1.14±0.05	$1.05\pm 0.05^{b}$	1.19±0.07	$1.15\pm 0.11^{b}$	0.88±0.03
GG	4.12±0.17	$6.42\pm 0.26^{\mathrm{ab}}$	5.97±0.62	13.34±0.79	9.20±0.39	1.19±0.05	$1.04\pm 0.05^{b}$	1.19±0.09	$1.33\pm 0.12^{a}$	0.89±0.03
*OLR1*										
AA	4.15±0.15	6.25±0.23	$6.02\pm 0.52^{b}$	13.45±0.74	9.31±0.34	1.16±0.05	1.07±0.05	1.20±0.07	1.25±0.11	0.90±0.02
AC	4.18±0.14	6.38±0.21	$6.59\pm 0.50^{a}$	13.63±0.68	9.62±0.32	1.18±0.04	1.05±0.04	1.15±0.06	1.21±0.10	0.89±0.03

W1 – 100 kg, W2 – 200 kg, W3 – 300 kg, W4 – 400 kg, W5 – 450 kg body weight; FCR – feed conversion rate (dry matter basis); TFCR – total feed conversion rate throughout the entire experimental period (dry matter basis); ADWG – average daily weight gain; TADG – total average daily weight gain throughout the entire experimental period. ${}^{a,b}$ Different superscripts within a column indicate significant difference.

**Table 8:** Estimates of additive and dominance effects of the markers showing significant associations in the present study.

Trait	Corresponding	Additive	Dominant	Overall
	markers	effect${}^{1,2}$	effect${}^{1,3}$	P value${}^{1,4}$
DTRW2	*LEP*	$-8.67^{***}$	-2.43	0.001
DTRW3	*LEP*	-7.32	8.09${}^{**}$	0.008
	*FABP4* 3691	-18.44	$-16.76^{*}$	0.037
	*GHR*	6.75	6.31${}^{*}$	0.034
DTRW4	*CAPN1* 316	-13.36	$-33.04^{**}$	0.005
DTRW5	*LEP*	$-10.91^{**}$	5.77	0.006
W2–W3 FP	*FABP4* 2834	$-4.37^{*}$	-0.02	0.015
	*LGB*	3.45	2.47${}^{*}$	0.047
	*GHR*	2.88	3.52${}^{**}$	0.006
W4–W5 FP	*GHR*	-3.45	9.25${}^{*}$	0.019
FW	*CAPN1* 316	21.66	18.13${}^{**}$	0.003
W2–W3 DMI	*CAST*	-13.89	16.67${}^{*}$	0.042
	*GHR*	17.23	15.66${}^{**}$	0.006
TDMI	*LEP*	-26.01	159.00${}^{*}$	0.018
	*IGF1*	55.50	$-94.51^{*}$	0.020
	*GHR*	155.20${}^{***}$	-40.50	0.000
W1–W2 DDMI	*FABP4* 3533	0.13	$-0.12^{**}$	0.004
W4–W5 DDMI	*MYF5*	0.01	0.39${}^{*}$	0.034
	*GHR*	0.16	$-0.48^{*}$	0.040
TDDMI	*LEP*	$-0.71^{***}$	-0.12	0.000
	*FABP4* 3533	-0.14	$-0.28^{*}$	0.011
	*IGF1*	0.14	$-0.19^{*}$	0.020
	*LGB*	-0.19	$-0.26^{*}$	0.012
W2–W3 FCR	*CAST*	-0.16	0.18${}^{*}$	0.023
	*GHR*	0.21	0.22${}^{**}$	0.004
W3–W4 FCR	*OLR1*${}^{5}$	–	–	0.024
W4–W5 FCR	*FABP4* 3533	-0.06	$-0.77^{*}$	0.031
TFCR	*LEP*	-0.18	0.19${}^{*}$	0.048
	*IGF1*	-0.42	0.30${}^{**}$	0.004
	*CAPN1* 316	-0.62	$-0.56^{***}$	0.001
W2–W3 ADWG	*FABP4* 2834	0.05${}^{*}$	-0.01	0.019
	*LGB*	$-0.04^{*}$	-0.02	0.036
	*GHR*	-0.04	$-0.03^{**}$	0.009
W4–W5 ADWG	*FABP4* 3533	0.03	0.13${}^{*}$	0.019
	*GHR*	0.06	$-0.13^{*}$	0.047
TADWG	*CAPN1* 316	0.05	0.04${}^{**}$	0.007

W1 – 100 kg, W2 – 200 kg, W3 – 300 kg, W4 – 400 kg, W5 – 450 kg body weight; DTR – days to reach; FP – fattening period; FW – final weight; DMI – dry matter intake; TDMI – total dry matter intake throughout the entire experimental period; DDMI – daily dry matter intake; TDDMI – total daily dry matter intake throughout the entire experimental period; FCR – feed conversion rate; TFCR – total feed conversion rate throughout the entire experimental period; ADWG – average daily weight gain; TADG – total average daily weight gain throughout the entire experimental period. ${}^{1}$ Least squares means for each genotype are shown in Tables 5–7. ${}^{2}$ Additive effect is estimated as the difference between the two homozygous means divided by 2 (Falconer and Mackay, 1996). ${}^{3}$ Dominance effect is estimated as the nonadditive genetic effects or the deviation of the heterozygote from the mean of the two homozygotes (Falconer and Mackay, 1996). ${}^{4}$ Overall P value for marker as a fixed genotype effect. ${}^{5}$ Only two genotypes were observed for *OLR1* marker, accordingly, additive and dominance effect could not be estimated. ${}^{*}$ P<0.05, ${}^{**}$ P<0.01, ${}^{***}$ P<0.001.

### 3.2 Marker associations

Levels of significance are reported in Table 4 for the effects of polymorphisms at the *LEP*, *FABP4*, *DGAT1*, *TG*, *IGF1*, *IGF1R*, *MYF5*, *LGB*, *CAPN1*, *CAST*, *GHR*, and *OLR1* genes on fattening performance traits in Holstein bulls. The association analysis revealed that all markers tested, with the exceptions of *CAPN1* 530, *IGF1R*, *TG*, and *DGAT1*, showed associations (at least one significant association) with respect to fattening performance, comprising 36 significant associations (P<0.05, P<0.01, P<0.001). The LSM and their respective SE obtained for the mentioned traits established are presented in Tables 5–7, whereas contributions of gene action, estimated as additive and dominance effects, are shown in Table 8. Results indicated that the marker *FABP4* 3691 was significantly associated with DTRW3 (P<0.05). Moreover, 2834 polymorphism at the same gene was effective in the W2–W3 interval with respect to FP and ADWG (P<0.05). *FABP4* 3533 marker affected W1–W2 DDMI, TDDMI, W4–W5 FCR, and W4–W5 ADWG at different levels of significance. *CAPN1* 316 was highly associated with DTRW4, FW, TFCR, and TADWG, while *CAST* was associated with W2–W3 DMI and FCR (P<0.05). Regarding the association analyses, the effectiveness of *LEP* and *GHR* markers are well demonstrated; and in this respect, these two markers seem to be strong candidates for various fattening performance traits in both evaluation of target-weight intervals and overall perspective, thereby affecting six and nine traits, respectively. Concerning *IGF1*, significant effects were observed for daily and total DMI (P<0.05) and TFCR (P<0.01). *MYF5* and *OLR1* markers were associated with W4–W5 DDMI and W3–W4 FCR, respectively; whereas *LGB* was significantly effective on FP and ADWG in the W2–W3 interval and TDDMI (P<0.05). There was no association between any of the tested SNPs with DTRW1, W1–W2 FP, W3–W4 FP, W1–W2 DMI, W3–W4 DMI, W4–W5 DMI, W2–W3 DDMI, and W3–W4 DDMI, nor was there any association with variation in W1–W2 FCR, W1–W2 ADWG, or W3–W4 ADWG (individual effects of the markers).

### 3.3 Genotypic interactions

The results of interaction analyses are reported in Tables 9 and 10. According to the results of analyses, significant associations were as follows:

a. *LEP* × *GHR* with DTRW2 (P<0.001), W1–W2 FP (P<0.01), W1–W2 DMI (P<0.01), W1–W2 FCR (P<0.001), W4–W5 FCR (P<0.05), TFCR (P<0.05), and W4–W5 ADWG (P<0.05);
b. *IGF1* × *LEP* with DTRW2 (P<0.001), DTRW3 (P<0.01), DTRW4 (P<0.001), W3–W4 FP (P<0.001), W4–W5 FCR (P<0.01), W4–W5 ADWG (P<0.01), and TADWG (P<0.001);
c. *FABP4* 3691 × *FABP4* 2834 with TFCR (P<0.05);
d. *FABP4* 3533 × *LEP* with DTRW4 (P<0.01).

Associations of *LEP* × *MYF5* with TFCR; *LEP* × *OLR1* with W4–W5 FP, TDMI, and W3–W4 ADWG; *IGF1* × *DGAT1* with DTRW5 were found to be statistically significant (P<0.05). However, these interactions were not considered because of the extremely unbalanced genotypic subsets (data not shown) in order to prevent unreliable results and they will not be discussed further.

**Table 9:** Least-square means and standard errors for the significant associations of genotypic interactions (*LEP* × *GHR* and *LEP* × *IGF1*) with related traits (n=296).

*LEP* × *GHR*								
Genotype	n	DTRW2${}^{***}$	W1–W2 FP${}^{**}$	W1–W2 DMI${}^{**}$	W1–W2 FCR${}^{***}$	W4–W5 FCR${}^{*}$	TFCR${}^{*}$	W4–W5 ADWG${}^{*}$
CCAA	25	$225.64\pm 9.56^{a}$	$100.03\pm 5.33^{\mathrm{ab}}$	$468.21\pm 19.05^{a}$	$4.68\pm 0.19^{a}$	$14.34\pm 0.96^{a}$	$9.85\pm 0.37^{a}$	$1.19\pm 0.11^{\mathrm{ab}}$
CCGA	9	$207.30\pm 12.60^{\mathrm{ab}}$	$95.61\pm 9.82^{\mathrm{abc}}$	$440.75\pm 34.91^{\mathrm{ab}}$	$4.41\pm 0.35^{\mathrm{ab}}$	$13.79\pm 0.75^{\mathrm{ab}}$	$9.44\pm 0.49^{\mathrm{ab}}$	$1.09\pm 0.14^{b}$
CCGG	9	$204.40\pm 13.80^{\mathrm{ab}}$	$77.44\pm 7.50^{c}$	$384.26\pm 25.50^{b}$	$3.84\pm 0.26^{b}$	$12.14\pm 0.95^{b}$	$9.36\pm 0.52^{\mathrm{ab}}$	$1.55\pm 0.15^{a}$
CTAA	28	$204.16\pm 9.65^{\mathrm{ab}}$	$87.25\pm 5.13^{\mathrm{bc}}$	$404.01\pm 18.42^{b}$	$4.04\pm 0.18^{b}$	$13.08\pm 0.79^{b}$	$9.47\pm 0.38^{\mathrm{ab}}$	$1.29\pm 0.12^{\mathrm{ab}}$
CTGA	9	$221.90\pm 13.90^{\mathrm{ab}}$	$93.25\pm 7.67^{\mathrm{abc}}$	$427.23\pm 27.28^{\mathrm{ab}}$	$4.27\pm 0.27^{\mathrm{ab}}$	$13.22\pm 1.18^{\mathrm{ab}}$	$10.21\pm 0.54^{a}$	$1.09\pm 0.15^{\mathrm{ab}}$
CTGG	8	$211.50\pm 15.60^{\mathrm{ab}}$	$95.67\pm 8.07^{\mathrm{abc}}$	$457.84\pm 28.77^{\mathrm{ab}}$	$4.58\pm 0.29^{\mathrm{ab}}$	$12.94\pm 1.03^{b}$	$10.53\pm 0.61^{a}$	$1.39\pm 0.16^{\mathrm{ab}}$
TTAA	143	$202.07\pm 7.71^{b}$	$90.51\pm 4.14^{\mathrm{abc}}$	$429.38\pm 14.96^{\mathrm{ab}}$	$4.29\pm 0.15^{\mathrm{ab}}$	$13.44\pm 0.63^{\mathrm{ab}}$	$9.51\pm 0.30^{a}$	$1.26\pm 0.09^{\mathrm{ab}}$
TTGA	50	$213.47\pm 8.80^{\mathrm{ab}}$	$96.31\pm 4.71^{\mathrm{abc}}$	$440.74\pm 16.94^{\mathrm{ab}}$	$4.41\pm 0.17^{\mathrm{ab}}$	$13.25\pm 0.72^{\mathrm{ab}}$	$9.22\pm 0.34^{\mathrm{ab}}$	$1.24\pm 0.11^{\mathrm{ab}}$
TTGG	15	$221.70\pm 11.10^{\mathrm{ab}}$	$105.97\pm 6.39^{a}$	$450.16\pm 23.45^{\mathrm{ab}}$	$4.50\pm 0.23^{\mathrm{ab}}$	$14.28\pm 0.82^{a}$	$8.48\pm 0.42^{b}$	$1.19\pm 0.12^{\mathrm{ab}}$
*IGF1* × *LEP*								
Genotype	n	DTRW2${}^{***}$	DTRW3${}^{**}$	DTRW4${}^{***}$	W3–W4 FP${}^{***}$	W4–W5 FCR${}^{**}$	W4–W5 ADWG${}^{**}$	TADWG${}^{***}$
CCCC	10	$257.20\pm 14.35^{a}$	$352.52\pm 17.81^{a}$	$550.01\pm 25.87^{a}$	$124.20\pm 24.83^{a}$	$15.23\pm 1.04^{a}$	$1.07\pm 0.15^{\mathrm{ab}}$	$1.08\pm 0.15^{c}$
CCCT	12	$200.71\pm 9.86^{b}$	$301.30\pm 12.83^{\mathrm{ab}}$	$462.90\pm 19.25^{c}$	$100.25\pm 8.39^{b}$	$13.02\pm 0.74^{\mathrm{ab}}$	$1.35\pm 0.11^{\mathrm{ab}}$	$1.09\pm 0.06^{c}$
CCTT	11	$229.51\pm 11.34^{\mathrm{ab}}$	$342.62\pm 14.62^{\mathrm{ab}}$	$550.63\pm 21.51^{a}$	$100.26\pm 9.54^{b}$	$14.33\pm 0.88^{\mathrm{ab}}$	$1.08\pm 0.12^{b}$	$1.33\pm 0.07^{\mathrm{ab}}$
CTCC	22	$197.62\pm 22.70^{b}$	$272.01\pm 37.92^{b}$	$491.12\pm 40.30^{\mathrm{abc}}$	$96.70\pm 11.80^{b}$	$13.50\pm 1.89^{\mathrm{ab}}$	$1.42\pm 0.29^{a}$	$1.49\pm 0.09^{a}$
CTCT	14	$212.73\pm 9.44^{\mathrm{ab}}$	$321.76\pm 12.42^{\mathrm{ab}}$	$484.83\pm 18.97^{\mathrm{bc}}$	$93.59\pm 8.25^{b}$	$13.14\pm 0.76^{\mathrm{ab}}$	$1.31\pm 0.12^{\mathrm{ab}}$	$1.05\pm 0.06^{c}$
CTTT	158	$193.52\pm 13.01^{b}$	$296.15\pm 18.92^{b}$	$489.65\pm 23.66^{\mathrm{abc}}$	$85.51\pm 11.9^{b}$	$14.96\pm 1.12^{\mathrm{ab}}$	$0.96\pm 0.15^{b}$	$1.17\pm 0.09^{\mathrm{abc}}$
TTCC	11	$215.73\pm 10.33^{\mathrm{ab}}$	$312.40\pm 13.91^{\mathrm{ab}}$	$480.80\pm 20.05^{\mathrm{bc}}$	$90.30\pm 9.01^{b}$	$12.47\pm 0.81^{b}$	$1.42\pm 0.12^{a}$	$1.03\pm 0.07^{c}$
TTCT	19	$208.19\pm 8.00^{b}$	$307.35\pm 10.72^{\mathrm{ab}}$	$487.34\pm 16.18^{\mathrm{bc}}$	$94.82\pm 7.01^{b}$	$13.79\pm 0.64^{\mathrm{ab}}$	$1.22\pm 0.09^{\mathrm{ab}}$	$1.10\pm 0.05^{c}$
TTTT	39	$203.44\pm 8.28^{b}$	$303.76\pm 10.93^{\mathrm{ab}}$	$479.45\pm 16.44^{\mathrm{bc}}$	$99.64\pm 7.39^{b}$	$14.11\pm 0.67^{\mathrm{ab}}$	$1.19\pm 0.10^{\mathrm{ab}}$	$1.18\pm 0.05^{\mathrm{bc}}$

W1 – 100 kg, W2 – 200 kg, W3 – 300 kg, W4 – 400 kg, W5 – 450 kg body weight; DTR – days to reach; FP – fattening period; DMI – dry matter intake; TFCR – total feed conversion rate throughout the entire experimental period (dry matter basis); ADWG – average daily weight gain; TADWG – total average daily weight gain throughout the entire experimental period. ${}^{a,b,c}$ Different superscripts within a column indicate significant difference. ${}^{*}$ P<0.05, ${}^{**}$ P<0.01, ${}^{***}$ P<0.001.

**Table 10:** Least-square means and standard errors for the significant associations of genotypic interactions (*FABP4* 3691 × *FABP4* 2834 and *FAP4* 3533 × *LEP*) with related traits (n=296).

*FABP4* 3691 × *FABP4* 2834${}^{*}$				*FAP4* 3533 × LEP${}^{**}$		
Genotype	n	TFCR		Genotype	n	DTRW4
AACC	3	$9.27\pm 1.31^{\mathrm{ab}}$		AACC	5	$406.15\pm 44.70^{b}$
AAGC	4	$8.26\pm 0.67^{b}$		AACT	5	$423.82\pm 38.01^{\mathrm{ab}}$
AAGG	4	$10.83\pm 0.59^{a}$		AATT	32	$453.33\pm 25.92^{\mathrm{ab}}$
GACC	7	$9.41\pm 0.61^{\mathrm{ab}}$		TACC	26	$471.35\pm 24.01^{a}$
GAGC	84	$9.41\pm 0.31^{\mathrm{ab}}$		TACT	25	$454.24\pm 29.22^{\mathrm{ab}}$
GAGG	34	$9.59\pm 0.37^{\mathrm{ab}}$		TATT	129	$422.16\pm 20.91^{\mathrm{ab}}$
GGCC	22	$9.51\pm 0.45^{\mathrm{ab}}$		TTCC	12	$426.54\pm 32.72^{\mathrm{ab}}$
GGGC	97	$9.26\pm 0.29^{\mathrm{ab}}$		TTCT	15	$438.63\pm 33.74^{\mathrm{ab}}$
GGGG	41	$9.79\pm 0.35^{\mathrm{ab}}$		TTTT	47	$465.82\pm 24.12^{a}$

TFCR – total feed conversion rate throughout the entire experimental period (dry matter basis); DTRW4 – days to reach 400 kg body weight. ${}^{a,b}$ Different superscripts within a column indicate significant difference. ${}^{*}$ P<0.05, ${}^{**}$ P<0.01.

## 4 Discussion

### 4.1 General perspectives

In the current study, several genes were selected based on their biological functions to be evaluated for association between polymorphisms and fattening performance. These genes were chosen because they have been shown to be involved in the regulation of appetite, lipid metabolism, or growth. Moreover, the effects of some functional candidate gene markers for milk production (e.g., *LGB* and *OLR1*) or meat quality (e.g., *CAPN1* and *CAST*) were tested for fattening performance traits because of their potential influence on growth and lipid metabolism as indicated by previous studies (Miquel et al., 2009; Fonseca et al., 2015). Impact of the selected genes seemed most likely to be related with animal body weight, weight gain, and feed efficiency, which are the crucial constituents of fattening performance evaluation (Sherman et al., 2008). SNPs within the *LEP*, *FABP4*, *DGAT1*, *TG*, *IGF1*, *IGF1R*, *MYF5*, *LGB*, *CAPN1*, *CAST*, *GHR*, and *OLR1* genes were examined for their effects on these traits in Holstein cattle. In the current study, we hypothesized that individual or combined effects of the genotypes at the selected genes may be effective on performance traits at different periods of animal growth and fattening. In this respect, the distinctive aspect of the evaluation presented in this paper, when compared to other cattle fattening performance studies, is the determination of genotype effects on reaching selected target body weights and the comparison of the traits among the intervals between them (see Sect. 2). Postnatal growth and fattening performance are under the polygenic inheritance. Genetic background of the animals, the existence of genotype × environment interactions, and linkage phase in the case of linked genes make the evaluation of performance traits much more complex (Pintos and Corva, 2011). The strong emphasis on genotypic effects expressed at different stages of an animal's life should be considered based on molecular aspects. For example, there is experimental evidence of a role for the calpain–calpastatin system in muscle cell migration and differentiation at early stages of development (Dedieu et al., 2004; Moyen et al., 2004; Barnoy et al., 2005). Another example is that, the concentration of leptin can be involved in food consumption, energy expenditure, and adipose tissue development (Buchanan et al., 2002; Lagonigro et al., 2003), which are fairly variable over different time periods of growth. On the other hand, it is well known that the genetic and phenotypic correlations among weights at different ages exist (Miquel et al., 2009). Taken altogether, evaluation of genetic marker effects with respect to different stages of development and fattening may provide novel aspects to fattening performance in livestock.

### 4.2 Genetic variability and population genetic indices

The present results showed a deviation from HWE for all the markers, except *IGF1R*, *CAPN1* 530, and *CAST*. Possible explanations for SNPs that showed deviation from HWE may be through population substructure. In this context, this disequilibrium might be due to the typical structure of dairy cow breeds, such as Holstein–Friesian, under intense selection that use artificial insemination with a few sires producing a large number of daughters. On the other hand, indirect selection for these loci from the selection for milk production should be considered when evaluating the Holstein breed (Lacorte et al., 2006). As pointed out by several authors, the markers that have MAF below 5 % should be excluded from association analyses to draw reliable conclusions (Bongiorni et al., 2012). Nevertheless, the highest frequency observed for the most common allele was 0.88 (*TG* marker); and accordingly, all the markers used in the current study can be evaluated as polymorphic loci. In genetic studies, population genetic indices (Ho, He, Ne, and PIC) play an important role in the assessment of population structure defined by genetic variation in a particular gene or genes. The levels of He may indicate clues in breeding characteristics of population; to give an example, the decrease in He can be caused as a result of eventual inbreeding. Besides, Ne expresses the effectiveness of loci allele impact in populations (Trakovicka et al., 2013). PIC values are the most common indices to determine the extent of the polymorphism of a marker. In this sense, the usefulness of a marker in segregation analysis is directly related to its level of polymorphism (Machado et al., 2010). PIC value classes are PIC > 0.50 (high polymorphism), 0.25 < PIC < 0.50 (moderate polymorphism), and PIC < 0.25 (low polymorphism) as suggested by Botstein et al. (1980). According to these criteria, the great majority of markers genotyped in the present population might be considered moderately polymorphic. However, regarding *TG* and *CAPN1* 530 markers, low values of indices were observed as follows: He < 0.30, Ne < 1.40, and PIC values of <0.25. On the other hand, concerning *FABP4* 3533, *DGAT1*, *IGF1*, and *CAST* markers, He values approached 0.50, whereas Ne values approached 2.00 in this study. The reason for the above-mentioned results is directly related to allele frequency distributions, which are known to vary between breeds and even between different populations of the same breed (Carvalho et al., 2012).

### 4.3 Individual marker effects on trait means

Results of this study show that SNP associations with cattle fattening performance traits, such as DMI, FCR, and ADWG, can be complex. This estimated complexity of fattening performance illustrates how SNP associations could differ when the target weights and stages of growth and fattening differ. To the knowledge of the authors, this is the first detailed report of associations between genotypic properties and fattening performance of Holsteins. The raw phenotypic data used for this study included 204 879 records (including daily feed consumption records of the individual animals). Genotypic analyses indicated that *LEP* A80V may be a potential marker for growth and fattening performance evaluation in cattle. In this context, animals with the TT genotype reached the target body weights of 200, 300, and 450 kg faster than alternative genotypes. Further, and more important, these animals reached the mentioned body weights with significantly lower TDMI and TDDMI (P<0.05 and P<0.001, respectively). This situation was substantiated by the evaluation of FCR values, since the TT animals had -0.36 and -0.37 kg kg${}^{-1}$ lower TFCR compared to CC and heterozygous animals, respectively. It is well known that, *LEP* gene encodes a protein hormone secreted from white adipose tissue that is involved in feed intake, appetite modulation, and energy metabolism, which may indicate its role in the growth processes (Kulig and Kmiec, 2009). Moreover, this gene exhibits a potent regulation through central and peripheral pathways and might affect body weight. Kulig and Kmiec (2009) reported that A80V polymorphism significantly affected the body weight at 210 days of age (P≤0.01) and the average daily gain between three and 210 days of age (P≤0.05) in Limousin breed with T as a desirable allele. Similarly, Nkrumah et al. (2006) indicated that the CC homozygous beef steers were characterized by a significantly lower daily gain compared with the heterozygous and TT animals. On the contrary, Ardicli et al. (2017a) reported that there was no significant association between the *LEP* A80V marker and fattening performance traits in Simmental bulls. Taken together, this study demonstrated that *LEP* A80V polymorphism may be highly associated with growth and fattening performance traits in Holsteins and TT genotype might contribute to faster growth (according to DTR target body weights) and improved FCR in Holstein cattle. Although *LEP* A80V is known as a famous candidate marker, knowledge about its associations with fattening performance is rather limited and the studies were mostly conducted on milk production traits. Therefore, further research on associations between the *LEP* and growth, or fattening traits in cattle, is necessary and may provide valuable contributions to selection programs.

BTA14 has been widely studied and the results indicated that it harbors many QTLs associated with fat metabolism. Several genetic markers associated with economically important traits, such as *FABP4*, *DGAT1*, and *TG*, are localized on this genomic region. *FABP4* is a leading candidate gene with an impact on lipid synthesis, feed intake, and growth (Yan et al., 2018). In the current study, polymorphisms in the *FABP4* gene were significantly associated with performance traits expressed at different stages of fattening in Holsteins. In this respect, *FABP4* 3691 was significantly associated with DTRW3 and animals with the AA genotype were characterized by decreased growth (P<0.05). AA animals reached the 300 kg body weight +35.20 and +36.88 days slower compared to GA and GG animals, respectively. In addition, the *FABP4* 2834 marker affected the FP and ADWG in the W2–W3 period (200–300 kg target body weights). Animals with the GG genotype exhibited higher ADWG (+0.09 and +0.05 kg day${}^{-1}$) and lower FP (-8.74 and -4.35 days) compared to CC and GC animals, respectively. The variation of another SNP in *FABP4*, g.3533T > A, was significantly associated with DDMI in the W1–W2 period (P<0.01) and TDDMI (P<0.05). The TT animals exhibited higher W1–W2 DDMI but lower TDDMI compared to alternative genotype carriers. Moreover, heterozygous genotype was associated with the lowest FCR but the highest ADWG values in the W4–W5 period. Even though it has been reported that *FABP4* plays an important role in cattle based on biological properties, especially fatness traits (Shin et al., 2012), it has not been securely suggested for the impact of variation on growth or fattening performance traits. Here, we present a probability of relationship between *FABP4* gene polymorphisms and cattle growth. To the best of our knowledge, no previously reported studies have detected such associations between *FABP4* genotypes and the above-mentioned performance traits, and hence the present results may provide novel aspects to this genomic region. Similar to the *FABP4 *gene, *DGAT1* and *TG* were located on BTA14, responsible for large effects on several economically important traits (Marques et al., 2008, 2009; Lee et al., 2013). However, there was no association between the polymorphisms of these genes and any of the phenotypic traits evaluated in the current study. Ardicli et al. (2017a) have indicated the same result, given that the *DGAT1* K232A is not an effective marker on fattening performance traits in Simmental bulls. The K232A and the C422T SNPs of the *DGAT1* and *TG* genes, respectively, have been shown to be associated with adjusted fat thickness, meat tenderness (Tait et al., 2014), and marbling (Burrell et al., 2004; Shin and Chung, 2007b). Nevertheless, adequate studies on the association of these markers with growth and fattening traits have not been widely performed, and thus further analyses in various breeds of cattle are needed to test possible relationships and to observe novel associations.

*IGF1* and its corresponding receptor *IGF1R* encode the hormones that are of basic constituents in the somatotrophic axis, and thus these genes play a key role in the regulation of the mammalian metabolism (Curi et al., 2005a). In this study, significant associations between *IGF1* C472T polymorphism and feed intake were observed. The TT genotype was characterized by the highest TDMI and TDDMI but the lowest TFCR. In this context, animals with the TT genotype had +0.32 and +0.27 kg day${}^{-1}$ higher values of TDDMI, while, -0.51 and -0.42 kg kg${}^{-1}$ lower TFCR compared to those with CT and CC genotypes. These findings agree in part with those reported by Siadkowska et al. (2006) who observed significant associations between the same genetic marker and feed intake or feed conversion for growth. These researchers suggested that animals carrying genotype BB (nucleotide CC) consumed less dry matter, crude protein, and energy per 1 kg body live weight gain than those of alternative genotypes. On the other hand, previous studies performed by Ge et al. (2001) and Curi et al. (2005a) have shown that *IGF1* C472T polymorphism (also known as *IGF1*/*Sna*BI) has significant effects on live weight and weight gain. However, these associations were not substantiated in the present study. For *IGF1R* G404T polymorphism, also known as *IGFIR*/*Taq*I, there was no significant association between the SNP and any of the performance traits in this study. Moody et al. (1996) and Curi et al. (2005a) indicated that this polymorphism is not very useful in studies on the identification of QTL in cattle, due to unbalanced allele frequencies. Moreover, Switonski (2002) suggested that BTA21, where the *IGF1R* is located, is not one of the most interesting for the localization of loci related to growth and carcass composition in beef cattle. These statements are in line with those obtained in the present study and are also confirmed for Holstein bulls.

A mutation of A/G in the intron 2 region of the *MYF5* gene has been reported to be associated with the various growth traits including weight at 12 months and ADWG (Chung and Kim, 2005); preweaning average daily gain and average daily gain on feed (Li et al., 2004); withers height and height at hip cross (Zhang et al., 2007). Here, we only report an association of the *MYF5* genotypes with W4–W5 DDMI (P<0.05). In this respect, heterozygous animals had +0.39 and +0.38 kg day${}^{-1}$ higher DDMI values compared to AA and GG animals, respectively. Extensive investigations have provided the detection of several polymorphisms in the *MYF5* gene in cattle. However, association analyses mostly revealed inconsistent results in different breeds or failed to demonstrate any relationship to production traits. Genotype–phenotype associations of the same genes in different lines or breeds may provide variable results because of their genetic constitutions. Moreover, evaluating the existence of interactions such as epistasis, pleiotropy, and genetic linkage should be taken into account when considering different combinations of the polymorphisms (Carvalho et al., 2012). The SNP studied here is located in an intron region, and thus it does not alter the amino acid sequence of the protein. However, introns have been shown to affect transcriptional efficiency (Zhang et al., 2007) and play an important role in gene regulation. Accordingly, there is still a need for further genetic studies about the *MYF5* effects on growth and fattening performance.

The polymorphism located in a coding region of the *LGB* gene (exon IV) showed significant associations with respect to FP and ADWG in the W2-W3 period and TDDMI (P<0.05). AA animals were characterized by the lowest values of W2–W3 FP, while the highest values of W2–W3 ADWG and TDDMI compared to alternative genotype carriers. The results obtained in the present study differ from those reported by Curi et al. (2005b) who did not observe any significant association between *LGB* genotypes and growth or carcass traits. One possible explanation of the lack of a significant association in their study may be through the genetic constitution of experimental animals and unbalanced genotypic frequencies (AA genotype frequency was very low). On the other hand, Tambasco et al. (2003) reported that, *LGB* × *GH* interaction was significantly effective on ADWG from weaning to yearling (P<0.05). The *LGB* gene has been reported to be an important factor in the evaluation of the milk production and quality traits. Moreover, this gene indicates a maternal ability in beef cattle and therefore represents a potential candidate gene for growth traits (Curi et al., 2005b). The effects of milk protein polymorphisms on growth or fattening traits have been limitedly investigated. To our knowledge, this is the first study indicating a possible connection between *LGB*/*Hae*III polymorphism and performance traits including DMI, and partially ADWG and FP. These findings may be useful to achieve novel aspects in evaluation of potential genetic markers with respect to livestock selection programs.

In the current study, *CAPN1* and *CAST *markers showed significant associations with performance traits. *CAPN1* G316A affected the DTRW4, FW, TFCR, and TADWG, while *CAST* was effective on DMI and FCR in the W2–W3 period. As pointed out by several authors, these genes are considered strong functional candidates for meat quality (Smith et al., 2000; Corva et al., 2007; Cheong et al., 2008; Bonilla et al., 2010; Curi et al., 2010). Moreover, there is fair evidence for an association between the telomeric end of BTA29, where the *CAPN1* is located, and performance traits including weaning weight, carcass weight, and feed efficiency (Casas et al., 2003; Miquel et al., 2009; Pintos and Corva, 2011). The present results demonstrated an association between the GG genotype of the *CAPN1* G316A marker and FW and TADWG (P<0.01), which can be evaluated as a strong dominance. At slaughter, animals carrying this genotype was +43.30 kg heavier than those animals with CC genotype. Moreover, GG animals had +0.08 kg day${}^{-1}$ higher ADWG compared to CC animals. These findings agree in part with those reported by Miquel et al. (2009) and Pintos and Corva (2011). Previous studies by some members of our study group have also shown that there is a strong association between *CAPN1* G316A marker and FW, given that the G is the favorable allele (Ardicli et al., 2017a, c). It is worth noting that allele C of this marker has been associated with more tender meat in several studies (Corva et al., 2007; Gill et al., 2009; Miquel et al., 2009). It is well known that the genetic and phenotypic correlations among weights at different ages are positive, and thus it is possible that meat tenderness shows an association with FW, and that selection for this marker may lead to changes in both traits and also in ADWG (Miquel et al., 2009). On the other hand, the observed results can be the consequence of an effect of tightly linked genes through calpain–calpastatin system. Concerning also the effect of *CAST* marker, it seems that both markers may be involved in some way in growth regulation. If so, as suggested by Pintos and Corva (2011), selection for growth rate and body size in many beef cattle breeds could explain, at least partially, the reported allele frequencies (frequency of G allele is highly dominant over C) in markers such as *CAPN1* G316A. Here, we present that a similar implementation may be considered for Holsteins.

The present study clearly demonstrated that the *GHR* S555G polymorphism is a strong candidate marker that influences performance traits in Holstein breed. Nine phenotypic traits including DTRW3, W2–W3 FP, W2–W3 DMI, W2–W3 FCR, W2–W3 ADWG, W4–W5 FP, W4–W5 DDMI, W4–W5 ADWG, and TDMI were significantly affected by this polymorphism at different levels of significance (Table 4). Results indicated that AA animals reached the 300 kg target body weight (DTRW3) faster than alternative genotype carriers (∼13 days). Moreover, these animals had the highest values for TDMI and W2–W3 ADWG. Heterozygous animals were characterized by a longer FP at both W2–W3 and W4–W5 periods and a higher FCR value in the W2–W3 period. On the other hand, animals with the GG genotype had higher DMI in the W2–W3, DDMI and ADWG in the W4–W5 compared to AA, and heterozygous animals. These findings suggest that it is important to consider the marker effects on different stages of animal growth. In other words, genes may mediate their effects on metabolism as the cattle grow. However, Ardicli et al. (2017a) reported that *GHR* S555G polymorphism was not associated with any of the fattening performance traits in Simmental bulls. In the literature, there is limited information about the association of the *GHR* S555G marker with fattening performance traits and present results suggest that focusing on this genomic region may be useful in improving these traits.

The association analysis involving the *OLR1* g.8232C > A (also denoted as C223A) polymorphism genotypes and production traits only considered genotypes AA and AC since the CC genotype was not present in this study. Results revealed that there was a significant association between this marker and W3–W4 FCR. Heterozygous animals had +0.57 kg kg${}^{-1}$ higher values of FCR in the W3–W4 period compared to AA animals. The *OLR1* gene plays a key role in low-density lipoprotein degradation as well as in glucose and lipid metabolism in liver (Khatib et al., 2006). As suggested by Fonseca et al. (2015), this effect is important for weaning weight adjusted to 210 days of age with which they observed a significant association (P<0.05). One possible connection between the *OLR1* marker and FCR may be due to metabolism-related properties as mentioned above.

In the evaluation of a genetic basis for quantitative traits, it is important to consider how genotypic interactions influence the phenotypic variations. The substitution of amino acids by SNPs may change protein structures, resulting in altered mRNA translation and protein synthesis mechanisms. This situation can influence the functional significance of the corresponding genes (Ardicli et al., 2017b). Broad genetic diversity in livestock provides the opportunity for the selection of animals with superior performance in specific desirable traits (Williams, 2005). Thus, molecular marker information and evaluation of highly effective SNPs can be of great use for identification of animals with high genetic value for complex traits, such as fattening performance, and the selection process can be conducted on younger animals, even before birth.

### 4.4 Combined effects of markers

Association analyses have routinely considered each locus separately in most QTL studies, and therefore these studies do not account for interaction between different loci. As suggested by Tambasco et al. (2003), interactions between marker loci should be more carefully investigated as such interactions may account for differences in genotype responses across populations and genetic backgrounds. Here, we present some novel genetic associations with respect to combined effects of the markers selected. In this sense, the present study indicates that *LEP* × *GHR* and *IGF1* × *LEP* interactions have significant effects on performance traits. Regarding the *LEP* × *GHR* interaction, CCAA was characterized as an unfavorable genotype with the highest values of DTRW2, W1–W2 FCR, W4–W5 FCR, and TFCR. Moreover, the CCCC genotype of *IGF1* × *LEP* was also associated with the unfavorable effects including the highest values of DTRW2, DTRW3, DTRW4, W3–W4 FP, and W4–W5 FCR, and the lowest TADWG (Table 9). Selecting animals with these genotypes may have an inadequate impact to commercial farms. Animals with the AAGC genotype of the *FABP4* 3691 × *FABP4* 2834 exhibited significantly lower means (8.26±0.67) for TFCR compared to alternative genotypes. In addition, animals with the AACC genotype of the *FAP4* 3533 × *LEP* reached the 400 kg target body weight faster than the other genotypes (Table 10). The above-mentioned genes are known to be the regulators of lipid metabolism, and hence interactions between them may provide useful clues for understanding the molecular mechanisms behind cattle fattening performance.

## 5 Conclusions

An extensive genetic evaluation of fattening performance was performed in Holstein bulls. The results of this study suggest that polymorphisms at the *LEP*, *FABP4*, *IGF1*, *MYF5*, *LGB*, *CAPN1*, *CAST*, *GHR*, and *OLR1* genes influence economically important traits, such as final weight, dry matter intake, feed conversion rate, and average daily gains. The results also demonstrate that SNP associations may differ when the target weights and stages of growth or fattening differ. Here, it should also be noted that genotypic interactions may provide valuable information about the evaluation of fattening performance traits. In this respect, novel associations were observed for analyses carried out with *LEP* × *GHR*, *IGF1* × *LEP*, *FABP4* 3691 × *FABP4* 2834, and *FAP4* 3533 × *LEP* interactions. Selecting animals with the favorable SNP genotypes may result in production of animals with higher fattening performance and meat yield. Most of these selected polymorphisms are located in exonic regions and they cause amino acid alterations. *FABP4* g.2834C > G and g.3533T > A, which are located on introns 1 and 2, respectively, do not change the amino acid sequence of the protein but it is possible that they influence gene splicing, binding of regulatory proteins during transcription, mRNA stability, or even translation. On the other hand, epigenetic factors should be considered when evaluating complex genotypic interventions such as the genetic basis of growth and fattening traits. As more molecular-based knowledge is available on gene function, it may become apparent how these SNPs are contributing to variation in these traits, especially at different stages of an animal's growth. Another important point is that effects of these markers should be verified in other cattle populations to confirm the present associations before they can be applied to marker-assisted selection. Consequently, if the results of this study are confirmed, application of these markers in assisted selection will improve breeding decisions and have the capability to evaluate traits that are difficult to measure, such as fattening performance.

## Supplement

10.5194/aab-62-9-2019-supplement The supplement related to this article is available online at: https://doi.org/10.5194/aab-62-9-2019-supplement.

## Data Availability

The original data are available upon request to the corresponding authors.

## References

[bib1.bib1] Alberti P, Panea B, Sanudo C, Olleta J, Ripoll G, Ertbjerg P, Christensen M, Gigli S, Failla S, Concetti S (2008). Live weight, body size and carcass characteristics of young bulls of fifteen European breeds. Livest Sci.

[bib1.bib2] Ardicli S, Dincel D, Samli H, Balci F (2017). Effects of polymorphisms at LEP, CAST, CAPN1, GHR, FABP4 and DGAT1 genes on fattening performance and carcass traits in Simmental bulls. Arch Anim Breed.

[bib1.bib3] Ardicli S, Samli H, Alpay F, Dincel D, Soyudal B, Balci F (2017). Association of single nucleotide polymorphisms in the FABP4 gene with carcass characteristics and meat quality in Holstein bulls. Ann Anim Sci.

[bib1.bib4] Ardicli S, Samli H, Dincel D, Soyudal B, Balci F (2017). Individual and combined effects of CAPN1, CAST, LEP and GHR gene polymorphisms on carcass characteristics and meat quality in Holstein bulls. Arch Anim Breed.

[bib1.bib5] Banos G, Woolliams J, Woodward B, Forbes A, Coffey M (2008). Impact of single nucleotide polymorphisms in leptin, leptin receptor, growth hormone receptor, and diacylglycerol acyltransferase (DGAT1) gene loci on milk production, feed, and body energy traits of UK dairy cows. J Dairy Sci.

[bib1.bib6] Barendse W, Bunch R, Thomas M, Armitage S, Baud S, Donaldson N (2004). The TG5 thyroglobulin gene test for a marbling quantitative trait loci evaluated in feedlot cattle. Animal Prod Sci.

[bib1.bib7] Barnoy S, Maki M, Kosower NS (2005). Overexpression of calpastatin inhibits L8 myoblast fusion. Biochem Biophys Res Commun.

[bib1.bib8] Bongiorni S, Mancini G, Chillemi G, Pariset L, Valentini A (2012). Identification of a short region on chromosome 6 affecting direct calving ease in Piedmontese cattle breed. PLoS One.

[bib1.bib9] Bonilla C, Rubio M, Sifuentes A, Parra-Bracamonte G, Arellano V, Mendez M, Berruecos J, Ortiz R (2010). Association of CAPN1 316, CAPN1 4751 and TG5 markers with bovine meat quality traits in Mexico. Genet Mol Res.

[bib1.bib10] Botstein D, White RL, Skolnick M, Davis RW (1980). Construction of a genetic linkage map in man using restriction fragment length polymorphisms. Am J Hum Genet.

[bib1.bib11] Buchanan FC, Fitzsimmons CJ, Van Kessel AG, Thue TD, Winkelman-Sim DC, Schmutz SM (2002). Association of a missense mutation in the bovine leptin gene with carcass fat content and leptin mRNA levels. Genet Sel Evol.

[bib1.bib12] Burrell DN, Moser G, Hetzel J, Mizoguchi Y, Hirano T, Sugimoto Y, Mengersen K (2004). Meta analysis confirms associations of the TG5 thyroglobulin polymorphism with marbling in beef cattle.

[bib1.bib13] Calo LL, McDowell RE, Dale Van Vleck L, Miller PD (1973). Genetic aspects of beef production among Holstein-Friesians pedigree selected for milk production. J Anim Sci.

[bib1.bib14] Carvalho TDD, Siqueira F, Torres Junior RADA, Medeiros SRD, Feijo GLD, Junior S, Blecha IMZ, Soares CO (2012). Association of polymorphisms in the leptin and thyroglobulin genes with meat quality and carcass traits in beef cattle. Rev Bras Zootec.

[bib1.bib15] Casas E, Shackelford SD, Keele JW, Stone RT, Kappes SM, Koohmaraie M (2000). Quantitative trait loci affecting growth and carcass composition of cattle segregating alternate forms of myostatin. J Anim Sci.

[bib1.bib16] Casas E, Shackelford SD, Keele JW, Koohmaraie M, Smith TP, Stone RT (2003). Detection of quantitative trait loci for growth and carcass composition in cattle. J Anim Sci.

[bib1.bib17] Cheong HS, Yoon DH, Park BL, Kim LH, Bae JS, Namgoong S, Lee HW, Han CS, Kim JO, Cheong IC, Shin HD (2008). A single nucleotide polymorphism in CAPN1 associated with marbling score in Korean cattle. BMC Genet.

[bib1.bib18] Chung E, Kim W (2005). Association of SNP marker in IGF-I and MYF5 candidate genes with growth traits in Korean cattle, Asian-Australas. J Anim Sci.

[bib1.bib19] Corva PM, Soria L, Schor A, Villarreal E, Cenci MP, Motter M, Mezzadra C, Melucci L, Miquel C, Paván E (2007). Association of CAPN1 and CAST gene polymorphisms with meat tenderness in Bos taurus beef cattle from Argentina. Genet Mol Biol.

[bib1.bib20] Corva PM, Fernez Macedo GV, Soria LA, Papaleo Mazzucco J, Motter M, Villarreal EL, Schor A, Mezzadra CA, Melucci LM, Miquel MC (2009). Effect of leptin gene polymorphisms on growth, slaughter and meat quality traits of grazing Brangus steers. Genet Mol Res.

[bib1.bib21] Curi RA, de Oliveira H, Silveira AC, Lopes C (2005). Association between IGF-I, IGF-IR and GHRH gene polymorphisms and growth and carcass traits in beef cattle. Livest Prod Sci.

[bib1.bib22] Curi RA, de Oliveira HN, Gimenes MA, Silveira AC, Lopes CR (2005). Effects of CSN3 and LGB gene polymorphisms on production traits in beef cattle. Genet Mol Biol.

[bib1.bib23] Curi RA, Palmieri DA, Suguisawa L, de Oliveira HN, Silveira AC, Lopes CR (2006). Growth and carcass traits associated with GH1/Alu I and POU1F1/Hinf I gene polymorphisms in Zebu and crossbred beef cattle. Genet Mol Biol.

[bib1.bib24] Curi RA, Chardulo LAL, Mason M, Arrigoni M, Silveira A, de Oliveira H (2009). Effect of single nucleotide polymorphisms of CAPN1 and CAST genes on meat traits in Nellore beef cattle (Bos indicus) and in their crosses with Bos taurus. Anim Genet.

[bib1.bib25] Curi RA, Chardulo LAL, Giusti J, Silveira AC, Martins CL, de Oliveira HN (2010). Assessment of GH1, CAPN1 and CAST polymorphisms as markers of carcass and meat traits in Bos indicus and Bos taurus–Bos indicus cross beef cattle. Meat Sci.

[bib1.bib26] Curi RA, Chardulo LAL, Arrigoni M, Silveira AC, de Oliveira H (2011). Associations between LEP, DGAT1 and FABP4 gene polymorphisms and carcass and meat traits in Nelore and crossbred beef cattle. Livest Sci.

[bib1.bib27] Dedieu S, Poussard S, Mazeres G, Grise F, Dargelos E, Cottin P, Brustis J-J (2004). Myoblast migration is regulated by calpain through its involvement in cell attachment and cytoskeletal organization. Exp Cell Res.

[bib1.bib28] Di Stasio L, Destefanis G, Brugiapaglia A, Albera A, Rolando A (2005). Polymorphism of the GHR gene in cattle and relationships with meat production and quality. Anim Genet.

[bib1.bib29] Falconer DS, Mackay TFC (1996). Introduction to quantitative genetics.

[bib1.bib30] Fonseca PDDS, de Souza FR, de Camargo GM, Gil FM, Cardoso DF, Zetouni L, Braz CU, Boligon AA, Branco RH, Lucia G (2015). Association of ADIPOQ. OLR1 and PPARGC1A gene polymorphisms with growth and carcass traits in Nelore cattle, Meta Gene.

[bib1.bib31] Fortes MR, Curi RA, Chardulo LA, Silveira AC, Assumpcao ME, Visintin JA, de Oliveira HN (2009). Bovine gene polymorphisms related to fat deposition and meat tenderness. Genet Mol Biol.

[bib1.bib32] Ge W, Davis M, Hines H, Irvin K, Simmen R (2001). Association of a genetic marker with blood serum insulin-like growth factor-I concentration and growth traits in Angus cattle. J Anim Sci.

[bib1.bib33] Gill JL, Bishop SC, McCorquodale C, Williams JL, Wiener P (2009). Association of selected SNP with carcass and taste panel assessed meat quality traits in a commercial population of Aberdeen Angus-sired beef cattle. Genet Sel Evol.

[bib1.bib34] Green MR, Sambrook J (2012). Isolation of high-molecular-weight DNA from mammalian cells using proteinase K and phenol. Molecular Cloning: A Laboratory Manual.

[bib1.bib35] Grisart B, Coppieters W, Farnir F, Karim L, Ford C, Berzi P, Cambisano N, Mni M, Reid S, Simon P (2002). Positional candidate cloning of a QTL in dairy cattle: identification of a missense mutation in the bovine DGAT1 gene with major effect on milk yield and composition. Genome Res.

[bib1.bib36] Hradecka E, Citek J, Panicke L, Rehout V, Hanusova L (2008). The relation of GH1, GHR and DGAT1 polymorphisms with estimated breeding values for milk production traits of German Holstein sires. Czech J Anim Sci.

[bib1.bib37] Juszczuk-Kubiak E, Rosochacki SJ, Wicinska K, Szreder TS (2004). A novel RFLP/AluI polymorphism of the bovine calpastatin (CAST) gene and its association with selected traits of beef. Anim Sci Pap Rep.

[bib1.bib38] Kalm E, Reinsch N, Xu N, Thomsen H, Looft C, Grupe S, Brockmann G, Kuehn C, Schwerin M, Leyhe B, Hiendleder S, Erhardt G, Medjugorac I, Russ I, Förster M, Brening B, Reinhard F, Reents R, Averdunk G (1998). Mapping quantitative trait loci on cattle chromosome 2, 5, 10, 16, 18 and 23. Anim Genet.

[bib1.bib39] Khatib H, Leonard S, Schutzkus V, Luo W, Chang Y (2006). Association of the OLR1 gene with milk composition in Holstein dairy cattle. J Dairy Sci.

[bib1.bib40] Kirkpatrick BW, Byla BM, Gregory KE (2000). Mapping quantitative trait loci for bovine ovulation rate. Mamm Genome.

[bib1.bib41] Kisacova J, Kubek A, Melus V, Canakyova Z, Rehout V (2009). Genetic polymorphism of Myf-5 and myostatin in Charolais breed. J Agrobiol.

[bib1.bib42] Koknaroglu H, Loy D, Wilson D, Hoffman M, Lawrence J (2005). Factors affecting beef cattle performance and profitability. Prof Anim Sci.

[bib1.bib43] Komisarek J, Dorynek Z (2009). Effect ofABCG2, PPARGC1A, OLR1 andSCD1 gene polymorphism on estimated breeding values for functional and production traits in Polish Holstein-Friesian bulls. J Appl Genet.

[bib1.bib44] Kulig H, Kmiec M (2009). Association between leptin gene polymorphisms and growth traits in Limousin cattle. Russ J Genet.

[bib1.bib45] Lacorte G, Machado M, Martinez M, Campos A, Maciel R, Verneque R, Teodoro R, Peixoto M, Carvalho M, Fonseca C (2006). DGAT1 K232A polymorphism in Brazilian cattle breeds. Genet Mol Res.

[bib1.bib46] Lagonigro R, Wiener P, Pilla F, Woolliams J, Williams J (2003). A new mutation in the coding region of the bovine leptin gene associated with feed intake. Anim Genet.

[bib1.bib47] Lee SH, Choi BH, Lim D, Gondro C, Cho YM, Dang CG, Sharma A, Jang GW, Lee KT, Yoon D, Lee HK, Yeon SH, Yang BS, Kang HS, Hong SK (2013). Genome-wide association study identifies major loci for carcass weight on BTA14 in Hanwoo (Korean cattle). PLoS One.

[bib1.bib48] Li C, Basarab J, Snelling W, Benkel B, Murdoch B, Hansen C, Moore S (2004). Assessment of positional candidate genes MYF-5 and IGF-1 for growth on bovine chromosome 5 in commercial lines of Bos taurus. J Anim Sci.

[bib1.bib49] Lisa C, Di Stasio L (2009). Variability of -calpain and calpastatin genes in cattle. Ital J Anim Sci.

[bib1.bib50] Lusk J (2007). Association of single nucleotide polymorphisms in the leptin gene with body weight and backfat growth curve parameters for beef cattle. J Anim Sci.

[bib1.bib51] Machado MA, Azevedo ALS, Teodoro RL, Pires MA, Peixoto MGC, de Freitas C, Prata MCA, Furlong J, da Silva MVG, Guimaraes SE (2010). Genome wide scan for quantitative trait loci affecting tick resistance in cattle (Bos taurus x Bos indicus). BMC Genom.

[bib1.bib52] Machado MB, Alencar MM, Pereira AP, Oliveira HN, Casas E, Coutinho LL, Regitano LC (2003). QTL affecting body weight in a candidate region of cattle chromosome 5. Genet Mol Biol.

[bib1.bib53] Maj A, Oprzadek J, Oprzadek A, Dymnicki E, Zwierzchowski L (2004). Polymorphism in the 5 noncoding region of the bovine growth hormone receptor gene and its association with meat production traits in cattle. Anim Res.

[bib1.bib54] Marques E, Schnabel RD, Stothard P, Kolbehdari D, Wang Z, Taylor JF, Moore SS (2008). High density linkage disequilibrium maps of chromosome 14 in Holstein and Angus cattle. BMC Genet.

[bib1.bib55] Marques E, Nkrumah J, Sherman E, Moore S (2009). Polymorphisms in positional candidate genes on BTA14 and BTA26 affect carcass quality in beef cattle. J Anim Sci.

[bib1.bib56] Miquel MC, Villarreal E, Mezzadra C, Melucci L, Soria L, Corva P, Schor A (2009). The association of CAPN1 316 marker genotypes with growth and meat quality traits of steers finished on pasture. Genet Mol Biol.

[bib1.bib57] Moody D, Pomp D, Barendse W (1996). Linkage mapping of the bovine insulin-like growth factor-1 receptor gene. Mamm Genome.

[bib1.bib58] Moore S, Li C, Basarab J, Snelling W, Kneeland J, Murdoch B, Hansen C, Benkel B (2003). Fine mapping of quantitative trait loci and assessment of positional candidate genes for backfat on bovine chromosome 14 in a commercial line of. J Anim Sci.

[bib1.bib59] Moyen C, Goudenege S, Poussard S, Sassi AH, Brustis J-J, Cottin P (2004). Involvement of micro-calpain (CAPN 1) in muscle cell differentiation. Int J Biochem Cell Biol.

[bib1.bib60] Mundan D, Gogebakan S, Ergun C, Kaban IH (2012). Evaluation of fattening performance of Holstein cattle at different initial weights under summer season conditions in the district of Silifke of Mersin province. J Anim Vet Adv.

[bib1.bib61] Murase T, Kume N, Kataoka H, Minami M, Sawamura T, Masaki T, Kita T (2000). Identification of soluble forms of lectin-like oxidized LDL receptor-1. Arterioscler Thromb Vasc Biol.

[bib1.bib62] Nkrumah JD, Li C, Yu J, Hansen C, Keisler DH, Moore SS (2005). Polymorphisms in the bovine leptin promoter associated with serum leptin concentration, growth, feed intake, feeding behavior, and measures of carcass merit. J Anim Sci.

[bib1.bib63] Nkrumah JD, Li C, Keisler DH, Sherman EL, Wang Z, Murdoch BM, Moore SS (2006). Polymorphisms in the leptin gene and their associations with performance, feed efficiency, and carcass merit of beef cattle.

[bib1.bib64] Oprzadek J, Flisikowski K (2003). Polymorphisms at loci of leptin (LEP), Pit1 and STAT5A and their association with growth, feed conversion. Anim Sci Pap Rep.

[bib1.bib65] Oztabak K, Toker NY, Un C, Akis I (2010). Leptin gene polymorphisms in native Turkish cattle breeds. Kafkas Univ Vet Fak Derg.

[bib1.bib66] Pfuhl R, Bellmann O, Kühn C, Teuscher F, Ender K, Wegner J (2007). Beef versus dairy cattle: a comparison of feed conversion, carcass composition, and meat quality. Arch Tierz.

[bib1.bib67] Pintos D, Corva PM (2011). Association between molecular markers for beef tenderness and growth traits in Argentinian angus cattle. Anim Genet.

[bib1.bib68] Sherman E, Nkrumah J, Murdoch B, Li C, Wang Z, Fu A, Moore S (2008). Polymorphisms and haplotypes in the bovine neuropeptide Y, growth hormone receptor, ghrelin, insulin-like growth factor 2, and uncoupling proteins 2 and 3 genes and their associations with measures of growth, performance, feed efficiency, and carcass merit in beef cattle. J Anim Sci.

[bib1.bib69] Shin SC, Chung ER (2007). Association of SNP marker in the leptin gene with carcass and meat quality traits in Korean cattle. Asian-Aust J Anim Sci.

[bib1.bib70] Shin SC, Chung E (2007). Association of SNP marker in the thyroglobulin gene with carcass and meat quality traits in Korean cattle. Asian-Aust J Anim Sci.

[bib1.bib71] Shin SC, Heo JP, Chung ER (2012). Genetic variants of the FABP4 gene are associated with marbling scores and meat quality grades in Hanwoo (Korean cattle). Mol. Biol Rep.

[bib1.bib72] Siadkowska E, Zwierzchowski L, Oprzadek J, Strzalkowska N, Bagnicka E, Krzyzewski J (2006). Effect of polymorphism in IGF-1 gene on production traits in Polish Holstein-Friesian cattle. Anim Sci Pap Rep.

[bib1.bib73] Smith TP, Casas E, Rexroad CE, Kappes SM, Keele JW (2000). Bovine CAPN1 maps to a region of BTA29 containing a quantitative trait locus for meat tenderness. J Anim Sci.

[bib1.bib74] Soria LA, Corva P, Huguet M, Mino S, Miquel M (2010). Bovine μ-calpain (CAPN1) gene polymorphisms in Brangus and Brahman bulls. J Basic Appl Sci.

[bib1.bib75] Stone R, Keele J, Shackelford S, Kappes S, Koohmaraie M (1999). A primary screen of the bovine genome for quantitative trait loci affecting carcass and growth traits. J Anim Sci.

[bib1.bib76] Switonski M (2002). Molecular genetics in beef cattle breeding – a review. Anim Sci Pap Rep.

[bib1.bib77] Tait RG, Shackelford SD, Wheeler TL, King DA, Keele JW, Casas E, Smith TP, Bennett GL (2014). CAPN1, CAST, and DGAT1 genetic effects on preweaning performance, carcass quality traits, and residual variance of tenderness in a beef cattle population selected for haplotype and allele equalization. J Anim Sci.

[bib1.bib78] Tambasco D, Paz C, Tambasco-Studart M, Pereira A, Alencar M, Freitas A, Coutinho L, Packer I, Regitano LDA (2003). Candidate genes for growth traits in beef cattle crosses Bos taurus x Bos indicus. J Anim Breed Genet.

[bib1.bib79] Trakovicka A, Moravcikova N, Kasarda R (2013). Genetic polymorphisms of leptin and leptin receptor genes in relation with production and reproduction traits in cattle. Acta Biochim Pol.

[bib1.bib80] (2018). Livestock Statistics [Internet].

[bib1.bib81] Ustuner H, Yalcintan H, Orman A, Ardicli S, Ekiz B, Gencoglu H, Kazoglu O (2017). Effects of initial fattening age on carcass characteristics and meat quality in Simmental bulls imported from Austria to Turkey. S Afr J Anim Sci.

[bib1.bib82] Vinsky M, Islam K, Chen L, Li C (2013). Association analyses of a single nucleotide polymorphism in the promoter of OLR1 with growth, feed efficiency, fat deposition, and carcass merit traits in hybrid, Angus and Charolais beef cattle. Can J Anim Sci.

[bib1.bib83] Williams JL (2005). The use of marker-assisted selection in animal breeding and biotechnology. Rev Sci Tech Off Int Epiz.

[bib1.bib84] Yan W, Zhou H, Hu J, Luo Y, Hickford JG (2018). Variation in the FABP4 gene affects carcass and growth traits in sheep. Meat Sci.

[bib1.bib85] Yeh FC, Yang RC, Boyle TB, Ye Z, Mao JX (2000). POPGENE, the user-friendly shareware for population genetic analysis, Molecular biology and biotechnology centre [Internet].

[bib1.bib86] Zhang R, Chen H, Lei C, Zhang C, Lan X, Zhang Y, Zhang H, Bao B, Niu H, Wang X (2007). Association between polymorphisms of MSTN and MYF5 genes and growth traits in three Chinese cattle breeds. Asian-Aust J Anim Sci.

