# Supplementary material for: Comprehensive assessment of candidate genes associated with fattening performance in Holstein–Friesian bulls
Source: Arch Anim Breed. 2019 Jan 21;62(1):9–32. doi: 10.5194/aab-62-9-2019 (PMC6852860; doi:10.5194/aab-62-9-2019)
Supplement: The supplement related to this article is available online at: https://doi.org/10.5194/aab-62-9-2019-supplement. [file aab-62-9-supplement.pdf]

Supplement of Arch. Anim. Breed., 62, 9–32, 2019
https://doi.org/10.5194/aab-62-9-2019-supplement
© Author(s) 2019. This work is distributed under
the Creative Commons Attribution 4.0 License.

Open Access
Archives Animal Breeding

Supplement of

Comprehensive assessment of candidate genes associated with fattening performance in Holstein–Friesian bulls

Sena Ardicli et al.

Correspondence to: Faruk Balci (fbalci@uludag.edu.tr)

The copyright of individual parts of the supplement might differ from the CC BY 4.0 License.

Table S1 Detailed information about the studied genes and single nucleotide polymorphisms (SNPs)

Gene	BTA	Localization	NCBI Gene ID	Genbank Accession Number	SNP Name*	SNP Localization	Allele	Amino Acid Alterations
LEP	4	92,436,922-92,453,653	280836	AF536174.1	A80V	Exon 3	C/T	Ala/Val
FABP4	14	44,676,542-44,681,059	281759	NC_007312.4	g.3691G>A	Exon 3	G/A	Val/Met
					g.2834C>G	Intron 1	C/G	-
					g.3533T>A	Intron 2	T/A	-
DGAT1	14	603,813-612,791	282609	AY065621	K232A**	Exon 8	K/A	Lys/Ala
TG	14	8,217,490-8,453,614	280706	X05380	C422T	5'UTR	C/T	-
IGF1	5	66,191,602-66,264,083	281239	AF210383	C472T	5'UTR	C/T	-
IGF1R	21	7,780,293-8,080,394	281848	U33122	G404T	Intron 12	A/B	-
MYF5	5q13	10,284,434-10,287,669	281335	M95684	g.1911A>G	Intron 2	A/G	-
*LGB****	11q28	103,255,824-103,264,276	280838	AC_000168.1	T5261C	Exon 4 / Intron 4	A/B	Asp/Val; Gly/Ala
CAPN1	29	43,400,333-43,427,397	281661	AF252504	G316A	Exon 9	C/G	Gly/Ala
					V530I	Exon 14	A/G	Val/Ile
CAST	7	96,033,978-96,167,151	281039	AF117813	S20T	Exon 1C/1D	G/C	Ser/Thr
GHR	20q17	31,868,624-32,178,311	280805	AF140284	S555G	Exon 10	G/A	Ser/Gly
OLR1	5	99,803,497-99,815,138	281368	NM_174132	g.8232C>A	3'UTR	A/C	-

LEP – leptin. *FABP4* – fatty acid binding protein 4. *DGAT1* – diacylglycerol acyltransferase 1. *TG* – thyroglobulin. *IGF1* – insulin-like growth factor 1. *IGF1R* – insulin-like growth factor receptor 1. *MYF5* – myogenic factor 5. *LGB* – beta-lactoglobulin. *CAPN1* – micromolar calcium-activated neutral protease 1. *CAST* – calpastatin. *GHR* – growth hormone receptor. *OLR1* – oxidized low-density lipoprotein receptor.

*Marker names were selected according to genomic regions or amino acid alterations according to HGSV.
**The two SNP in the DGAT1 gene lie immediately adjacent to one another in exon 8, and the two alleles at this locus are AA and GC, encoding lysine (K) and alanine (A) at the amino acid position 232.
***Also known as progestagen-associated endometrial protein (*PAEP*).

Table S2 Estimates of variance components of the markers showing significant associations

Trait	Corresponding Markers	Additive Effect[1,2]	Dominant Effect[1,3]	Presence of Overdominance[1,4]	Overall *P* value[1,5]
**DTRW2**	*LEP*	-8.67***	-2.43		0.001
**DTRW3**	*LEP*	-7.32	8.09**		0.008
	FABP4 3691	-18.44	-16.76*		0.037
	GHR	6.75	6.31*		0.034
**DTRW4**	*CAPN1* 316	-13.36	-33.04**	+	0.005
**DTRW5**	*LEP*	-10.91**	5.77		0.006
**W2-W3 FP**	*FABP4* 2834	-4.37*	-0.02		0.015
	LGB	3.45	2.47*		0.047
	GHR	2.88	3.52**		0.006
**W4-W5 FP**	*GHR*	-3.45	9.25*	+	0.019
**FW**	*CAPN1* 316	21.66	18.13**		0.003
**W2-W3 DMI**	*CAST*	-13.89	16.67*		0.042
	GHR	17.23	15.66**		0.006
**TDMI**	*LEP*	-26.01	159.00*		0.018
	IGF1	55.50	-94.51*	+	0.020
	GHR	155.20***	-40.50		0.000
**W1-W2 DDMI**	*FABP4* 3533	0.13	-0.12**		0.004
**W4-W5 DDMI**	*MYF5*	0.01	0.39*	+	0.034
	GHR	0.16	-0.48*	+	0.040
**TDDMI**	*LEP*	-0.71***	-0.12		0.000
	FABP4 3533	-0.14	-0.28*	+	0.011
	IGF1	0.14	-0.19*	+	0.020
	LGB	-0.19	-0.26*	+	0.012
**W2-W3 FCR**	*CAST*	-0.16	0.18*		0.023
	GHR	0.21	0.22**		0.004
**W3-W4 FCR**	*OLR1*[6]	–	–		0.024
**W4-W5 FCR**	*FABP4* 3533	-0.06	-0.77*	+	0.031
**TFCR**	*LEP*	-0.18	0.19*		0.048
	IGF1	-0.42	0.30**		0.004
	CAPN1 316	-0.62	-0.56***		0.001
**W2-W3 ADWG**	*FABP4* 2834	0.05*	-0.01		0.019
	LGB	-0.04*	-0.02		0.036
	GHR	-0.04	-0.03**		0.009
**W4-W5 ADWG**	*FABP4* 3533	0.03	0.13*	+	0.019
	GHR	0.06	-0.13*		0.047
**TADWG**	*CAPN1* 316	0.05	0.04**		0.007

W1 – 100 kg, W2 – 200 kg, W3 – 300 kg, W4 – 400 kg, W5 – 450 kg target body weight. DTR – days to reach. FP – fattening period. FW – final weight. DMI – dry matter intake. TDMI – total dry matter intake throughout the entire experimental period. DDMI – daily dry matter intake. TDDMI – total daily dry matter intake throughout the entire experimental period. FCR – feed conversion rate. TFCR – total feed conversion rate throughout the entire experimental period. ADWG – average daily weight gain. TADG – total average daily weight gain throughout the entire experimental period.

[1]Least squares means for each genotype are shown in Tables 5-7.
[2]Additive effect is estimated as the difference between the 2 homozygous means divided by 2 (a) (Falconer and Mackay, 1996).
[3]Dominance effect is estimated as the nonadditive genetic effects or the deviation of the heterozygote from the mean of the 2 homozygotes (d) (Falconer and Mackay, 1996).
[4]The degree of dominance may be expressed as d/a. If there is over dominance, d is greater than + a or less than – a (Falconer and Mackay, 1996).
[5]Overall *P* value for marker as a fixed genotype effect.
[6]Only two genotypes were observed for *OLR1* marker, accordingly, additive and dominance effect could not be estimated.
**P*< 0.05, ***P*<0.01, ****P*<0.001
